# The *Caenorhabditis elegans* Kinesin-3 Motor UNC-104/KIF1A Is Degraded upon Loss of Specific Binding to Cargo

**DOI:** 10.1371/journal.pgen.1001200

**Published:** 2010-11-04

**Authors:** Jitendra Kumar, Bikash C. Choudhary, Raghu Metpally, Qun Zheng, Michael L. Nonet, Sowdhamini Ramanathan, Dieter R. Klopfenstein, Sandhya P. Koushika

**Affiliations:** 1 National Centre for Biological Sciences, Tata Institute of Fundamental Research, Bangalore, India; 2 Department of Anatomy and Neurobiology, Washington University School of Medicine, St. Louis, Missouri, United States of America; 3 Center for Molecular Physiology of the Brain, Georg August Universität Göttingen, Göttingen, Germany; 4 Drittes Physikalisches Institut, Göttingen, Germany; Stanford University School of Medicine, United States of America

## Abstract

UNC-104/KIF1A is a Kinesin-3 motor that transports synaptic vesicles from the cell body towards the synapse by binding to PI(4,5)P_2_ through its PH domain. The fate of the motor upon reaching the synapse is not known. We found that wild-type UNC-104 is degraded at synaptic regions through the ubiquitin pathway and is not retrogradely transported back to the cell body. As a possible means to regulate the motor, we tested the effect of cargo binding on UNC-104 levels. The *unc-104(e1265)* allele carries a point mutation (D1497N) in the PI(4,5)P_2_ binding pocket of the PH domain, resulting in greatly reduced preferential binding to PI(4,5)P_2_ *in vitro* and presence of very few motors on pre-synaptic vesicles *in vivo*. *unc-104(e1265)* animals have poor locomotion irrespective of *in vivo* PI(4,5)P_2_ levels due to reduced anterograde transport. Moreover, they show highly reduced levels of UNC-104 *in vivo*. To confirm that loss of cargo binding specificity reduces motor levels, we isolated two intragenic suppressors with compensatory mutations within the PH domain. These show partial restoration of *in vitro* preferential PI(4,5)P_2_ binding and presence of more motors on pre-synaptic vesicles *in vivo*. These animals show improved locomotion dependent on *in vivo* PI(4,5)P_2_ levels, increased anterograde transport, and partial restoration of UNC-104 protein levels *in vivo*. For further proof, we mutated a conserved residue in one suppressor background. The PH domain in this triple mutant lacked *in vitro* PI(4,5)P_2_ binding specificity, and the animals again showed locomotory defects and reduced motor levels. All allelic variants show increased UNC-104 levels upon blocking the ubiquitin pathway. These data show that inability to bind cargo can target motors for degradation. In view of the observed degradation of the motor in synaptic regions, this further suggests that UNC-104 may get degraded at synapses upon release of cargo.

## Author Summary

The cell body and the synapse in a neuron are often separated by significant distance, which is spanned by the axon connecting the two. Transport of various cargoes along the axonal highway is very important for neuronal function. The regulation of this complex process is not well understood. Using the *Caenorhabditis elegans* model system, we have demonstrated for the first time the fate of a motor after it carries its cargo to the synapse from the cell body. We show that the UNC-104 motor, which carries pre-synaptic vesicles to the synapse, is degraded once it gets there. Moreover, our genetic studies show evidence that loss of cargo binding targets the motor for degradation, suggesting an attractive mechanism for the regulation of motors at the synapse. Our study opens up several further questions, such as the mechanism of motor degradation, and has significant implications for regulation of cargo transport.

## Introduction

Transport of pre-synaptic vesicles from the neuronal cell body to the synapse is an essential process to ensure that the nerve terminals can effectively participate in synaptic transmission [1], [2]. This transport is a regulated process that occurs primarily using the Kinesin-3 family motor UNC-104, Imac, KIF1A and KIF1Bβ, respectively, in the model systems *C. elegans, Drosophila,* mouse and humans [3]-[9]. In *C. elegans*, mutants in *unc-104* have locomotory defects that arise from the absence of transport of synaptic vesicles, leading to reduced synaptic transmission at neuromuscular junction synapses [3], [10].

Molecular motors in neurons such as UNC-104 are thought to bind to their cargoes in the cell body of the neuron, get transported along microtubule tracks to synapses and release their cargo upon reaching the synapse [2]. It has been proposed that upon release of cargo the motor gets either inactivated or degraded [11], thus suggesting cargo binding and cargo release as possible means to regulate motor levels. UNC-104 recognizes its cargo by binding PI(4,5)P_2_ present on the carrier vesicle via its PH domain [12] and its mammalian orthologue in addition uses other proteins to recognize cargo [13].

Several effects of cargo binding on the Kinesin-3 family motors have been shown. Cargo binding by a chimeric Kinesin-3 leads to aggregation of the motor on the cargo surface and improved processivity of the chimera [14], [15]. Mutations in the cargo-binding PH domain of UNC-104 that do not bind PI(4,5)P_2_ efficiently have also been suggested to affect processivity of the motor [12], [14]. Further, it has been proposed that UNC-104 dimerizes upon cargo binding [14]. The mammalian KIF1A has recently been reported to exist in a dimeric autoinhibited state from which it is released upon cargo binding [16], [17], showing that while the orthologues behave differently, they are both regulated by cargo binding. Similarly another motor, Kinesin-1, is maintained in an inactive folded state [18] and is activated by binding to regulatory molecules/cargo adaptors. Simultaneous binding by both JIP1 and Fez1 activates Kinesin-1 and allows the motor to bind microtubules [19].

Cargo release has also been postulated to play important roles in motor regulation [20]. Motors involved in anterograde axonal transport such as Kinesin-1, Kinesin-3/KIF1A and heterotrimeric Kinesin are all thought be regulated after releasing cargo at the synapse. All three motors, although transported robustly in the anterograde direction to the synapse, are not efficiently retrogradely transported [6], [21]-[23]. These observations have led to the hypothesis that once these motors release cargo at synapses, they are largely degraded, thus maintaining directionality of axonal transport [11].

We sought to test this hypothesis for the *C. elegans* UNC-104 motor protein. To do so, it is necessary to address the following two questions. 1) Does the motor get degraded at the synapse? 2) Does the motor get degraded once there is no binding to the cargo? To answer the first question, we established that the wild type motor is degraded in synaptic regions and that it does not return to the cell body from the synapse. We further showed that the degradation near the synapse takes place through the ubiquitin pathway. To address the second question, we studied the effect of lack of cargo binding on the *C. elegans* UNC -104 motor protein. For this we used a mutant UNC-104 motor and showed that it has greatly reduced ability to preferentially bind PI(4,5)P_2_ *in vitro* as well as greatly reduced presence on pre-synaptic vesicles *in vivo*. We found that this leads to almost total loss of the motor *in vivo*, even though the motor still retains the ability to bind other lipids. The relationship between ability to bind cargo and motor levels was verified by analyzing two intragenic suppressors of the original mutation in the PH domain. The suppressors only moderately reduce the ability to preferentially bind PI(4,5)P_2_ and we see that UNC-104 levels are partially restored. All three PH domain variants of the motor are degraded via the ubiquitin pathway in synapse rich regions of the animal. A triple mutant reversing the effect of one of the suppressor mutations again does not preferentially bind PI(4,5)P_2_ *in vitro*, does not provide behavioural rescue and does not show expression of UNC-104 *in vivo*. These findings, together with the observed degradation of wild type UNC-104 in synaptic regions, suggest that the synaptic vesicle motor UNC-104 is degraded upon release from pre-synaptic vesicles near the synapse.

## Results

### Wild-type UNC-104 is degraded at synapses

**Figure 1:**
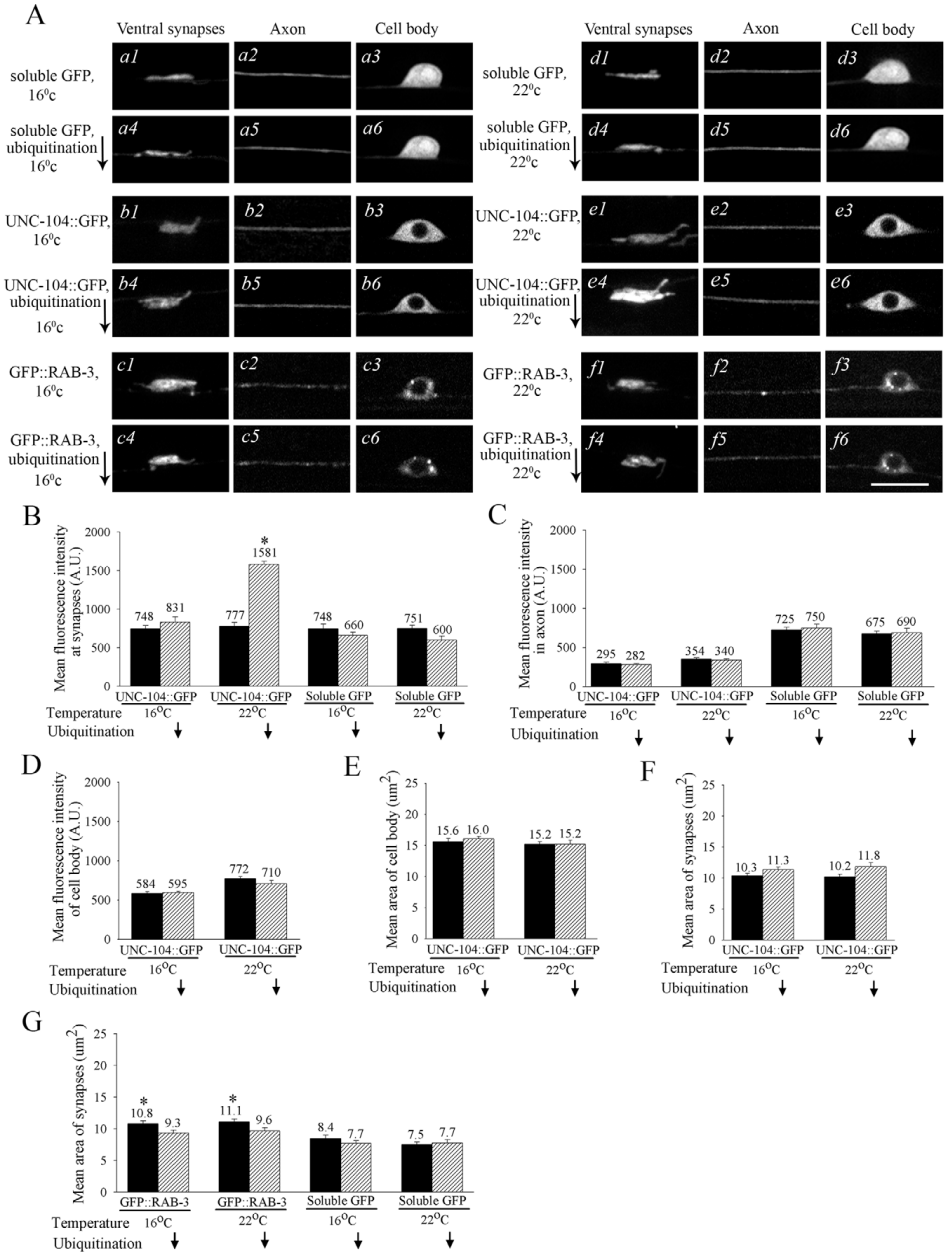
Degradation of UNC-104::GFP in mechanosensory neurons. (A) Effect of reduced ubiquitination (using the mutant *uba-1*) on sub-cellular UNC-104 levels. Expression levels of different transgenes in *uba-1(it129ts)* background in mechanosensory neurons. Left: 16°C (permissive) and right: 22°C (restrictive). Upper panel of each set is the transgene in a wild type background and the lower panel is the transgene in a *uba-1(it129ts)* mutant background. a1-a6, d1-d6: Ventral synapses, axon and cell body of animals expressing soluble GFP (*zdIs5*). b1-b6, e1-e6: Ventral synapses, axon and cell body of animals expressing UNC-104::GFP (*jsIs1111*). c1-c6, f1-f6: Ventral synapses, axon and cell body of animals expressing GFP::RAB-3 (*jsIs821*). Only animals expressing UNC-104::GFP show a prominent increase in fluorescent signal in synaptic regions of animals in which ubiquitination is reduced at 22°C. (B) Mean fluorescent intensity per pixel in arbitrary units (A.U.) of UNC-104::GFP and soluble GFP in wild type and *uba-1(it129ts)* background grown at 16°C and 22 °C. UNC-104::GFP intensity per pixel nearly double in the *uba-1(it129ts)* background at 22°C compared to the wild type background in synaptic regions (n = 25-35, *p = 10^-5^). (C, D) Mean fluorescent intensity per pixel in arbitrary units of UNC-104::GFP and soluble GFP in wild type and *uba-1(it129ts)* background grown at 16°C and 22°C in the axon (C) and cell body (D). (E, F) Mean area of the mechanosensory neuron cell body (E) and synapses (F) measured using UNC-104::GFP. (G) Mean area of the posterior mechanosensory neuron synapses measured using soluble GFP and GFP::RAB-3 in wild type and *uba-1(it129ts)* background grown at 16°C and 22°C. A small change in synapse area as measured by GFP::RAB-3 was seen in *uba-1(it129ts)* at both 16°C and 22°C (n = 30, p = 0.03). No such change was seen when using soluble GFP *zdIs5* as the marker. All data represented as mean ± SEM and n = 25-35 in all cases. Scale bar: 10 µm.

To determine whether the UNC-104/KIF1A motor is degraded at synapses we used a transgenic line over-expressing UNC-104::GFP in the six mechanosensory neurons of *C. elegans*. We examined the posterior neurons (PLM) whose morphology and synaptic locations are very well defined [24]. Further, a C-terminal UNC-104::GFP fusion provides functional rescue and its localization is similar to that of endogenous UNC-104 [25] suggesting that the addition of GFP does not impair the motor's *in vivo* function or localization. In a wild type background the UNC-104::GFP is present in the cell body, neuronal process and at synaptic regions (Figure 1A: b1-b3).

To determine whether UNC-104 is degraded we crossed the transgenic strain expressing UNC-104::GFP into a temperature sensitive *uba-1(it129ts)* mutant. *uba-1* encodes the only *C. elegans* E1 ubiquitin activating enzyme [26]. This activation is an early and essential step in the ubiquitin-degradation pathway. Consequently in *uba-1* animals ubiquitin-mediated degradation is reduced. At the lower growth temperature of 16°C the expression of UNC-104::GFP in *uba-1* animals is not significantly different from wild type in the cell body, neuronal process or at synaptic regions (Figure 1A: b1-b6, 1B). However, at the restrictive temperature of 22°C the expression of UNC-104::GFP significantly increases in synaptic regions (Figure 1A: e1,e4, 1B). The expression remains largely unchanged in the axon and in the cell body, although our method may not be sensitive to small changes in protein levels, especially in the narrow geometry of the neuronal process (Figure 1A: e2,e5, 1C, Figure S4H).

To confirm that the morphology of the mechanosensory neuron (including its synapses) is relatively unaffected in *uba-1* animals, we examined the localization and levels of soluble GFP and of the synaptic vesicle marker GFP::RAB-3 [27]-[29]. No alteration in expression levels of soluble GFP or GFP::RAB-3 was observed in the synapses, cell body or axon in *uba-1* animals (Figure 1A: d1-d6, f1-f6, 1C, 1D, Figure S4F, S4G, S4H). Compared to wild type, no changes were observed in the area and intensity of GFP in synaptic regions marked either by soluble GFP or by GFP::RAB-3 in *uba-1* animals, with the exception of a modest decrease observed in synaptic area marked by GFP::RAB-3 at the restrictive temperature (Figure 1A: d1-d6,f1-f6, 1B, 1C, 1G). This exception is consistent with the known importance of degradation for synapse formation in mechanosensory neurons [30]. Taken together these data suggest that development of the mechanosensory neurons and their synapses are not greatly altered in *uba-1(it129ts)* while there are significant effects on the levels of expression of the synaptic vesicle motor UNC-104 at synaptic regions.

**Figure 2:**
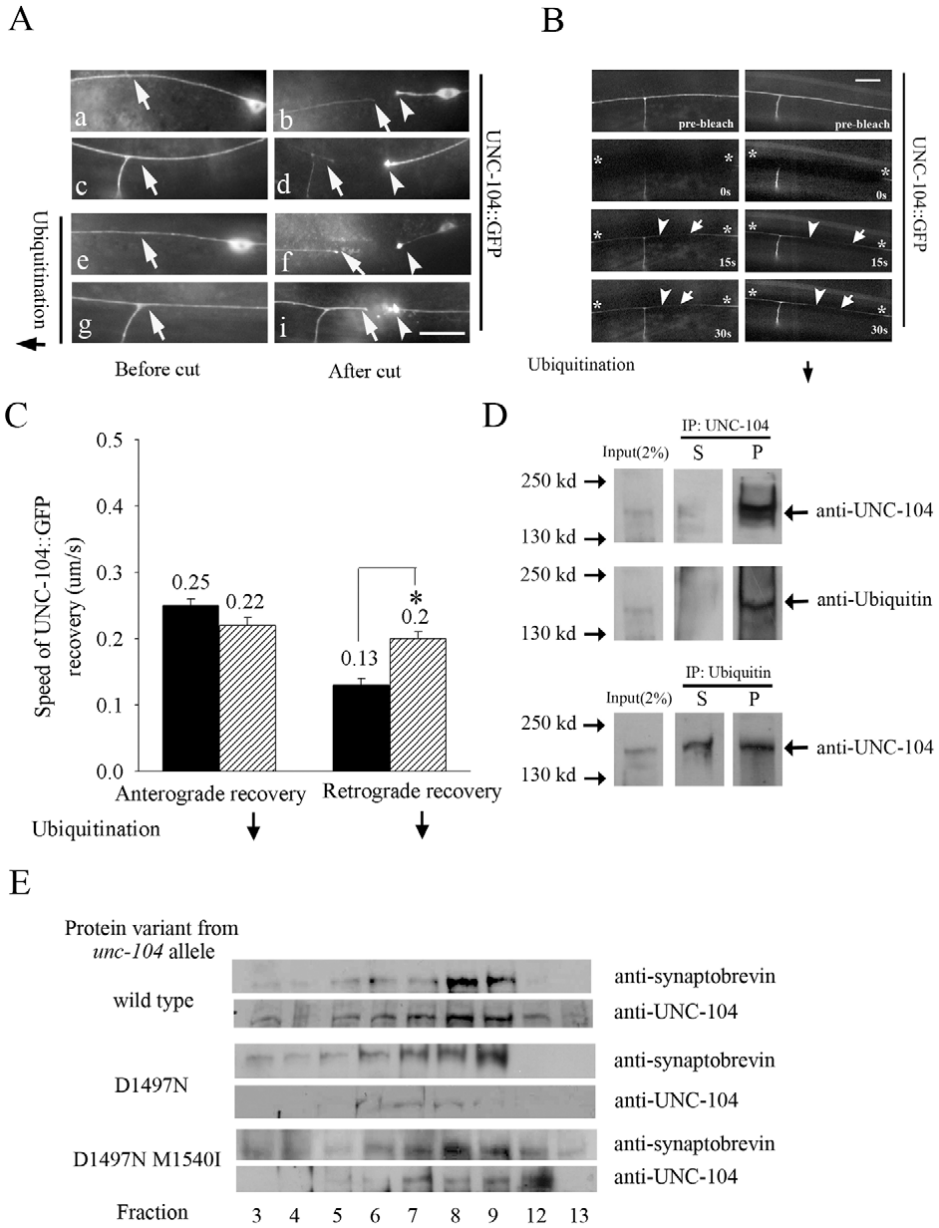
UNC-104 transport in mechanosensory neurons and co-sedimentation with pre-synaptic vesicles. (A) Laser microsurgery of UNC-104::GFP expressed in mechanosensory neurons (*jsIs1111*) upon block in ubiquitination using *uba-1(it129ts)*. (a,c,e,g) Arrow points to representative levels of UNC-104::GFP in the axon in wild type (a,c) and *uba-1* (e,g) animals. (b,d,f,i) The main neuronal process was cut 20 µm away from the cell body or 20 µm before the synaptic branch that ends in synapses. The UNC-104::GFP motor accumulated only in the proximal cut end after 1 hour (b, d arrowhead) and is greatly reduced in the distal neuronal process (b,d arrow). Upon axotomy in *uba-1* animals, UNC-104::GFP motor increased significantly in distal neuronal process (f,i arrowhead and arrow). Scale bar: 10 µm. (n = 15-20 animals) (B,C) Fluorescence recovery after photo bleaching of UNC-104::GFP in mechanosensory neurons upon block in ubiquitination using *uba-1(it129ts)*. Bleached area, anterograde recovery front and retrograde recovery front are marked by star, arrow and arrowhead respectively. The retrograde recovery of UNC-104::GFP is faster in *uba-1(it129ts)* than in wild type. Rate of recovery of UNC-104::GFP is represented as velocity of the UNC-104::GFP front in wild type and *uba-1(it129ts).* Rate of retrograde recovery of UNC-104::GFP is significantly increased in *uba-1* animals. Data represented as mean ± SEM (n = 30, *p<10^-4^). (D) Immunoprecipitation using anti-UNC-104 antibody (upper two panels) and anti-ubiquitin antibody (lower panel). Immunoprecipitation using anti-UNC-104 and subsequent western blot is probed with anti-UNC-104 and anti-ubiquitin. Immunoprecipitation using anti-ubiquitin and subsequent western blot is probed with anti-UNC-104. Input: 2% of lysate used for immunoprecipitation, S: supernatant after immunoprecipitation, P: immunoprecipitate. (E) Co-sedimentation of UNC-104 and pre-synaptic vesicles. Presence of pre-synaptic vesicles assayed using anti-synaptobrevin antibodies in the genotypes wild type, *unc-104(e1265)* and *unc-104(e1265tb120).* The alleles *unc-104(e1265)*, *unc-104(e1265tb107)* and *unc-104(e1265tb120)* are labeled in the figure by the respective protein changes they encode, namely D1497N, D1497N R1501Q and D1497N M1540I.

The above observations also suggest that UNC-104 may get degraded directly through attachment of ubiquitin (8 kDa) molecules to the motor. To test if UNC-104 is ubiquitinated we immunoprecipitated the endogenous UNC-104 motor (approximately 200 kDa) from a mixed-stage *C. elegans* extract. Western blot analysis of immunoprecipitated UNC-104 motor showed that the same band of about 200 kDa was recognized by both the anti-UNC-104 and the anti-ubiquitin antibodies (Figure 2D). Further, western analysis of the immunoprecipitate obtained using anti-ubiquitin and probed with anti-UNC-104 showed an approximately 200 kDa band, which migrates identically to the endogenous UNC-104 motor (Figure 2D). However, unlike the immunoprecipitation with the anti-UNC-104, immunoprecipitation using anti-ubiquitin showed the presence of UNC-104 in the supernatant. This signal may rise from UNC-104 molecules that are not ubiquitinated. Our observations suggest that UNC-104 can be ubiquitinated *in vivo.* Further, the data imply that UNC-104 transports synaptic vesicles to the synapse and upon reaching that location UNC-104 is degraded through the ubiquitin pathway, possibly through direct ubiquitination of the endogenous motor.

### UNC-104 is not retrogradely transported from synapses back to the cell body

Our observation that the motor reaching the synapse gets degraded predicts that there would be little retrograde transport of UNC-104 from the synapse back to the cell body. To test this hypothesis we carried out a transport assay by laser microsurgery of the mechanosensory neuron. We had observed that 1 hour after axotomy, cargoes such as GFP::RAB-3 and SNB-1::GFP accumulate on both sides of the cut site [31]. By contrast, wild type UNC-104::GFP accumulates only in the proximal region, i.e., at the end of the cut that is attached to the cell body (Figure 2A: b,d arrowhead). The distal end shows no accumulation of UNC-104::GFP, corroborating the hypothesis. Further the distal axon shows much lower level of UNC-104::GFP than the same region of the uncut axon (Figure 2A: b,d arrow). This reduction could result from UNC-104 in the distal axon being degraded after reaching the synapse. UNC-104::GFP has been shown in the seconds time-scale to undergo microscopic motion in both anterograde and retrograde directions but with a significant anterograde bias [25], which may result in overall bulk flow of the motor also being biased towards synaptic regions. Our results show that additionally, degradation of the motor in the synaptic region confers a macroscopic directionality to the movement of the motor. To further test this explanation, we carried out laser axotomy in *uba-1* animals and also did bleach recovery experiments to assess UNC-104 motor flow.

*uba-1* animals, one hour after laser axotomy of mechanosensory neurons expressing UNC-104::GFP, showed robust levels of UNC-104::GFP at the proximal regions (Figure 2A: f,i arrow) and significant levels in the distal regions as well (Figure 2A: e-i arrowhead). Thus upon blocking ubiquitination, UNC-104 is capable of macroscopic retrograde movement. To further confirm predicted trends in macroscopic motor movement in an uninjured neuron, we carried out a bleach recovery experiment of UNC-104::GFP in both wild type and *uba-1* animals. In either genotype, the UNC-104 motor recovers in both anterograde and retrograde directions in the time frame of seconds (Figure 2B). In wild type, the anterograde recovery is faster than the retrograde recovery, consistent with prior observations of anterograde bias of the microscopic movements of UNC-104::GFP (Figure 2C) [25]. Further, supporting our hypothesis, we observed that the retrograde recovery front moved faster in *uba-1* animals compared to wild type, while the anterograde recovery in the two genotypes did not differ significantly (Figure 2B, 2C).

These observations show that in wild type, very little of the anterograde motor UNC-104 is transported back to the cell body from the synapse. By contrast, when ubiquitin-mediated degradation is blocked, there is significant retrograde transport of the motor from the synapse towards the cell body, likely due to increased UNC-104 levels at synapses.

### The *unc-104(e1265)* allele encodes a D1497N change in its PH domain, leading to reduced ability to bind synaptic vesicle cargo

After establishing that the UNC-104 motor is degraded at synapses, we wished to study a possible mechanism for this process. One hypothesis is that once the motor gets to the synapse, it releases cargo and is then targeted for degradation [11], suggesting that degradation of the motor is linked to its being unbound to cargo. We decided to test this by studying the fate of the UNC-104 motors in a series of alleles that either strongly or moderately alter the ability of the motor to bind cargo through its PH domain.

**Figure 3:**
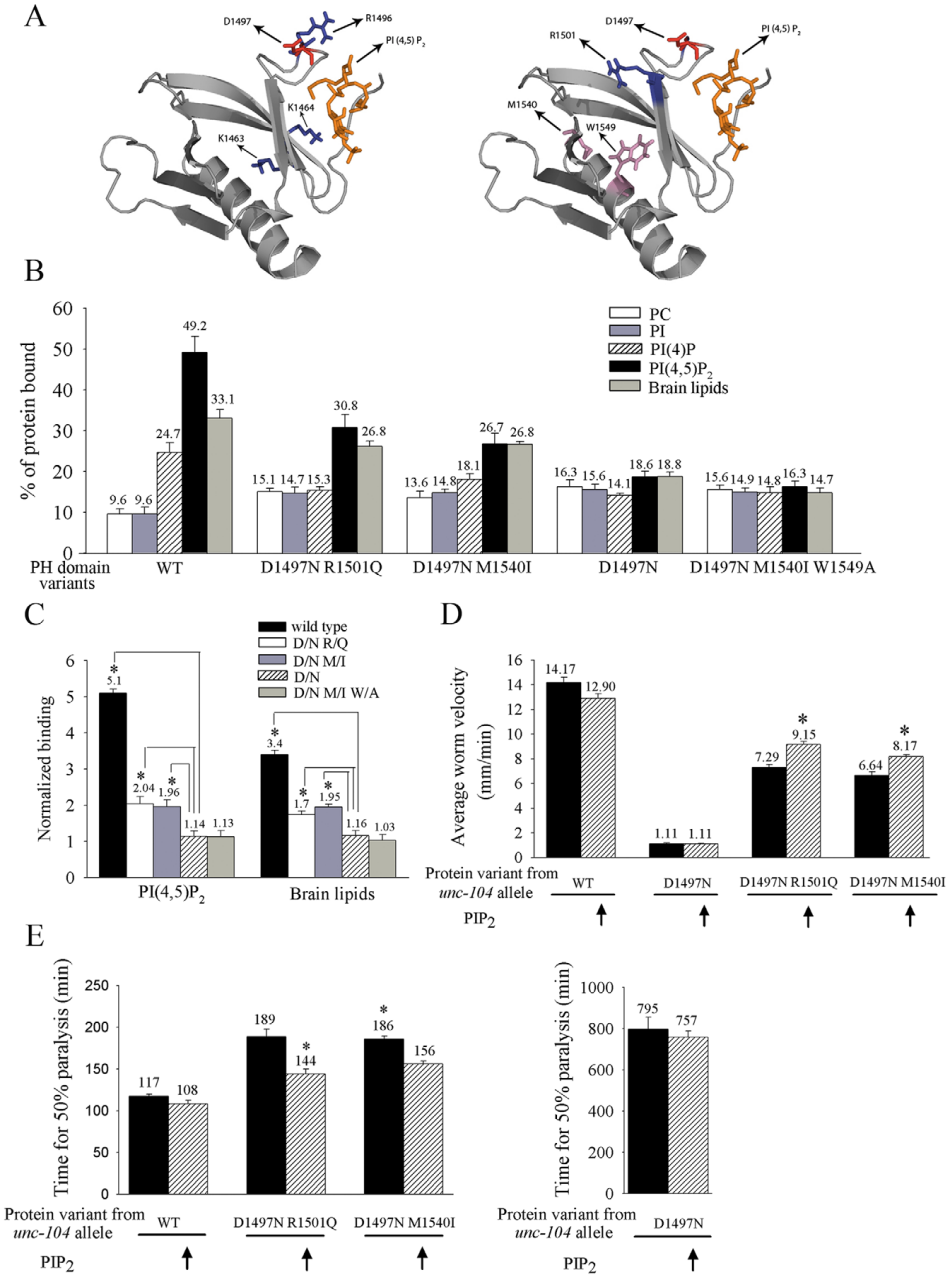
Binding of UNC-104 variants *in vitro* to PI(4,5)P_2_ and their *in vivo* sensitivity to PI(4,5)P_2_. (A) Homology model of the UNC-104 Pleckstrin homology (PH) domain docked with phosphatidylinositol-4,5-bisphosphate (PI(4,5)P_2_). Left: The original lesion in *unc-104(e1265),* D1497N, is marked along with the three residues (KK1463/4, R1496) known to be important for binding lipids. Note that the D1497N is a surface residue that lies in the same PI(4,5)P_2_ binding pocket as the other three residues. Right: Positions of the two residues that are altered in the intragenic suppressors *unc-104(e1265tb107)* (having the R1501Q lesion) and *unc-104(e1265tb120)* (having the M1540I lesion) along with the original lesion D1497N. Blue indicates basic residues; red indicates acidic residues and pink indicates mildly acidic residues. (B) Percentage binding of the UNC-104 PH domain variants to various lipids *in vitro*. All data represented as mean ± SD and obtained from four independent experiments assayed in triplicates. (C) Normalized binding of PI(4,5)P_2_ and brain lipids. Both D1497N and D1497N M1540I W1549A have very little specific binding to PI(4,5)P_2_ or brain lipids when normalized to PC binding. However the D1497N R1501Q and D1497N M1540I variants bind significantly better than D1497N to PI(4,5)P_2_. Data represented as mean ± SD (*p<0.005). PC – Phosphatidylcholine, PI(4,5)P_2_ – Phosphatidyl inositol-4,5-bisphosphate. (D, E) Responsiveness of *unc-104(e1265)*, *unc-104(e1265tb107)* and *unc-104(e1265tb120)* to increase in PI(4,5)P_2_ levels *in vivo* in all neurons. *gqIs25* over-expresses Type I PIP kinase (*ppk-1*) phosphatidylinositol-4-phosphate 5′ kinase in neurons and increases PI(4,5)P_2_ levels by 40% *in vivo*. (D) Locomotion of the various *C. elegans* strains. Wild type and *gqIs25* animals move well. Worm locomotion is unchanged in *unc-104(e1265)* upon increase in PI(4,5)P_2_. Locomotion is significantly improved in *unc-104(e1265tb107)* and *unc-104(e1265tb120)* when PI(4,5)P_2_ levels are increased (*p<10^-5^). n = 30 animals in all experiments. All data represented as mean ± SEM. (E) Aldicarb resistance of the various *C. elegans* strains as a measure of cholinergic transmission. Aldicarb inhibits acetylcholine esterase in *C. elegans* and causes hyperstimulation of the muscle and thus paralysis. Aldicarb resistance is unchanged in *unc-104(e1265)* upon increase in PI(4,5)P_2_. Time for paralysis is reduced in *unc-104(e1265tb107)* and *unc-104(e1265tb120)* when PI(4,5)P_2_ levels are increased (*p<0.001). n = 30 animals, done three times independently. Data represented as the average time (mean ± SEM) taken for 50% of the animals to be completely paralyzed. The alleles *unc-104(e1265)*, *unc-104(e1265tb107)* and *unc-104(e1265tb120)* are labeled in the figure by the respective protein changes they encode, namely D1497N, D1497N R1501Q and D1497N M1540I.

We first attempted to identify a pre-existing allele affecting cargo binding by sequencing several *unc-104* alleles (Figure S1A). Of these, *unc-104(e1265)*, a canonical allele, showed a single amino acid change D1497 to N in the PH domain (Figure S1A, S1B). To test participation of the highly conserved residue D1497 in binding PI(4,5)P_2_, we built a homology model of the UNC-104 PH domain using the crystal structure of the closest orthologue in the database, the protein DAPP1/PHISH (Figure 3A, Figure S1C) [32], [33]. The residues (KK1463/4 and R1496), known to be important for lipid binding [12], are respectively, 12 Å/16 Å and 3.8 Å from D1497N (Figure 3A). Thus the residue D1497 is on the surface of the PH domain in a region known to be important in binding PI(4,5)P_2_. Further, on docking the ligand PI(4,5)P_2_ on to the homology model using GRAMM [34] we observed that in 40% of the models it preferentially binds to the region juxtaposed to the D1497, R1496 and KK1463/4 residues (Figure 3A). The next two most common models (25%, 20%) identified for ligand docking do not show proximity to residues known to be important in PH domain-PI(4,5)P_2_ interactions.

To directly test the role of the D1497N mutation encoded by the *unc-104(e1265)* allele (which encodes the protein UNC-104(D1497N)) in binding to PI(4,5)P_2_, we carried out an *in vitro* liposome binding assay. The wild type UNC-104 PH domain binds preferentially to PI(4,5)P_2_, PI(4)P and brain lipids (Figure 3B) [12]. By contrast, the UNC-104 PH domain with the D1497N residue greatly reduces the preferential affinity for PI(4,5)P_2_, PI(4)P and brain lipids (Figure 3B, 3C). However the binding to both PC and PI increases compared to the wild type PH domain (Figure 3B). This suggests that the D1497N PH domain variant likely retains the ability to bind lipids even though the preferential binding to PI(4,5)P_2_ is highly decreased.

For further confirmation, we tested whether increasing PI(4,5)P_2_ *in vivo* provided functional rescue. In the transgenic line *gqIs25,* which over-expresses the PI(4,5)P_2_ biosynthetic enzyme *ppk-1* in neurons, PI(4,5)P_2_ levels are increased by 40% *in vivo* [35]. We tested functional rescue of transport using a locomotory behavioural assay and an aldicarb resistance assay. These assays depend on the release of neurotransmitter filled vesicles at synapses [36] that have been transported by the UNC-104 motor. (See materials and methods for the inverse relationship between synaptic transmission and paralysis induced by the acetylcholine esterase inhibitor aldicarb.) In *unc-104* mutants, vesicles at synaptic regions are greatly reduced [3], resulting in animals that are nearly immobile due to reduced synaptic transmission and are greatly resistant to paralysis induced by aldicarb (Figure 3D, 3E) [10]. Wild type and PI(4,5)P_2_ over-expressing animals have robust locomotion and are highly sensitive to aldicarb (Figure 3D, 3E). In *unc-104(e1265)* animals over-expressing PI(4,5)P_2_, there is no improvement in locomotory behaviour or sensitivity to aldicarb when compared to *unc-104(e1265)* animals (Figure 3D, 3E). Thus the protein encoded by the *unc-104(e1265)* allele with the D1497N lesion is insensitive to PI(4,5)P_2_ levels *in vivo*.

We wished to confirm that the reduced ability of the D1497N PH domain to bind PI(4,5)P_2_ *in vitro* results in correspondingly reduced ability of the UNC-104(D1497N) motor to bind to its synaptic vesicle cargo *in vivo*. For this we prepared pre-synaptic vesicles from *unc-104(e1265)* and wild type animals. Both genotypes have nearly identical levels of synaptic vesicles as assayed by the vesicle marker synaptobrevin (Figure 2E). At the same time, the vesicles prepared from *unc-104(e1265)* animals have very low amounts of UNC-104 present on them when compared to vesicles prepared from wild type animals (Figure 2E).

Thus UNC-104(D1497N), encoded by *unc-104(e1265)*, loses its ability to bind to PI(4,5)P_2_ *in vitro*, has greatly reduced presence of the mutant motor on its vesicular cargo *in vivo* and shows loss of synaptic vesicle transport irrespective of PI(4,5)P_2_ levels *in vivo*. This suggests that the motor encoded by the *unc-104(e1265)* allele is unable to transport synaptic vesicles through its inability to bind to its cargo.

### Intragenic suppressors of *unc-104(e1265)* improve synaptic protein localization and behaviour

To ameliorate the effects of *unc-104(e1265)* and to improve *in vivo* cargo binding, we screened ∼40,000 genomes in a behavioural suppressor screen using EMS mutagenesis. We identified four independent intragenic suppressors within the PH domain that improved the locomotory behaviour of *unc-104(e1265)* (Figure 3D). Of these, three alter the same residue, M1540I, while the other suppressor was an alteration at the highly conserved residue R1501 to Q1501 (Figure S1A, S1B). The three hits that result in the M1540I change were independently isolated at different times and did not always have the same nucleotide change (Figure S1A).

**Figure 4:**
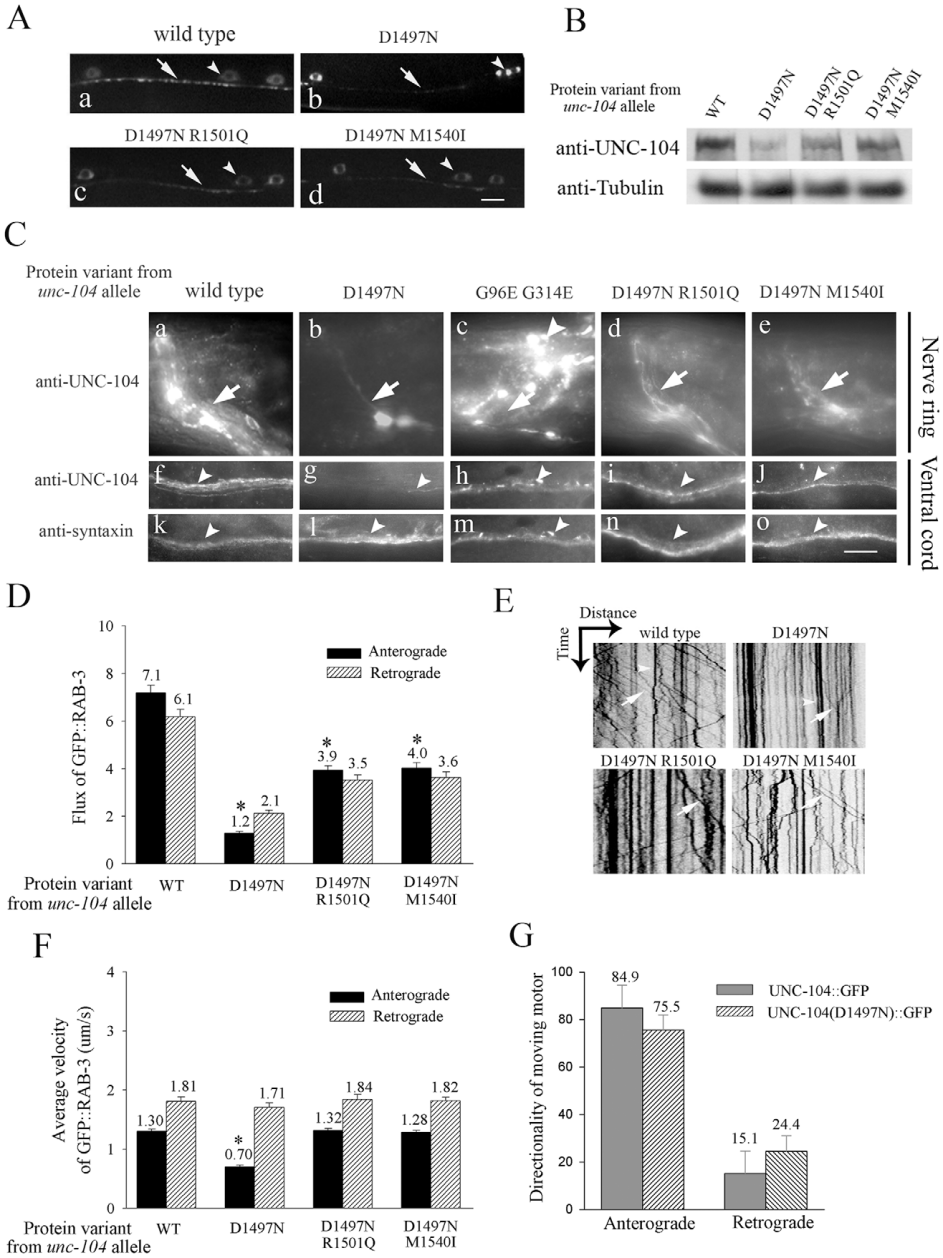
Phenotypic characterization of the *unc-104* allelic series. (A) Cargo molecules marked by synaptobrevin-1::GFP in motor neurons (*juIs1)* in wild type (a), *unc-104(e1265)* (b), *unc-104(e1265tb107)* (c) and *unc-104(e1265tb120)* (d). In all the images, the arrowhead points to the cell body and the arrow to the puncta at the neuromuscular junctions. Intensity and numbers of puncta along the process reflect the numbers of synaptic vesicles at motor neuron synapses. Scale bar: 10 µm. (B) Western blot analysis. Monoclonal anti-UNC-104 was used against wild type, *unc-104(e1265)* and its intragenic suppressors. As a control the same blot was probed with anti-tubulin. (C) anti-UNC-104 immunoreactivity using MAb 25H11. All experiments were done simultaneously and unsaturated images taken at identical exposures. UNC-104 is present in high levels in the nerve ring (arrow) and in the ventral cord (arrowhead) in wild type animals (a,f) but is greatly reduced in *unc-104(e1265)* (b,g) and partially restored in *unc-104(e1265tb107)* (d,i) and *unc-104(e1265tb120)* (e,j). Although UNC-104 is mis-localized no reduction in levels is seen in *unc-104(rh43)*, another allele with a lesion in the motor domain (c,h). Arrowhead in c,h points to a large number of cell bodies in the nerve ring and ventral cord region. Immunoreactivity to syntaxin that marks all neurons is unchanged in all *unc-104* alleles that encode PH domain variants (k,l,m,n,o). Scale bar: 10 µm. (D) Anterograde and Retrograde flux of GFP::RAB-3 in mechanosensory neurons. These data are obtained using movies, examples of which are provided in Videos S1, S2, S3. The number of particles moving to the synapse is highest in wild type animals and is significantly reduced in *unc-104(e1265)* (*p = 10^-19^). Anterograde flux is partially restored in the intragenic suppressors *unc-104(e1265tb107)* and *unc-104(e1265tb120)* compared to *unc-104(e1265)* (*p = 10^-15^, 10^-14^ respectively). Anterograde flux in *unc-104(e1265tb107)* and *unc-104(e1265tb120)* continues to be significantly lower than in wild type animals (*p = 10^-12^, 10^-11^ respectively). Retrograde flux parallels the anterograde trends in all genotypes. (E) Representative kymographs of various genotypes. A kymograph shows distance moved by GFP::RAB-3 particles (X-axis) over time (Y-axis). Anterogradely moving GFP::RAB-3 containing vesicles are indicated by arrows. Arrowheads point to stationary particles. (F) Anterograde and retrograde velocity of GFP::RAB-3 in wild type, *unc-104(e1265)* and intragenic suppressors [*unc-104(e1265tb107)*, *unc-104(e1265tb120)*]. Only the anterograde velocity in *unc-104(e1265)* shows any reduction (*p = 10^-29^) while all other measurements do not differ significantly from wild type. (G) Movement of UNC-104::GFP and UNC-104(D1497N)::GFP motors. Both wild type and mutant motors show a similar anterograde bias in movement. All data represented as mean ± SEM and collected from 13-15 animals. The alleles *unc-104(e1265)*, *unc-104(e1265tb107), unc-104(e1265tb120)* and *unc-104(rh43)* are labeled in the figure by the respective protein changes they encode, namely D1497N, D1497N R1501Q, D1497N M1540I and G96E G314E.

We wished to determine if the suppressors, *unc-104(e1265tb107)* and *unc-104(e1265tb120)* (respectively encoding the proteins UNC-104(D1497N R1501Q) and UNC-104(D1497N M1540I)), improved behaviour by altering synaptic vesicle distribution. We carried out an aldicarb resistance assay and also directly observed the distribution of a synaptic vesicle protein in motor neurons. The intragenic suppressors are less resistant to aldicarb compared to *unc-104(e1265)* (Figure 3E, Figure S2C). This indicates that both intragenic suppressors have greater release of acetylcholine at synapses. Consistent with these observations, we found that synaptobrevin-1::GFP (SNB-1::GFP), a synaptic vesicle protein marker transgenically expressed in motor neurons, accumulates largely in cell bodies rather than at synapses of *unc-104(e1265)* animals (Figure 4A: b) [24], [37] and the number of muscle arms connecting with synapses is greatly reduced (Figure S2A, S2B) [38]. In both intragenic suppressors the accumulation of SNB-1::GFP in cell bodies is greatly reduced and correspondingly more SNB-1::GFP is present at synapses and the number of muscle arms increases significantly (Figure 4A: c,d, Figure S2A, S2B).

Another synaptic vesicle marker GFP::RAB-3 [27], [28] behaves identically to SNB-1::GFP in mechanosensory neurons. In *unc-104(e1265)* the marker GFP::RAB-3 accumulates in the cell body with nearly no protein present at synapses. In both intragenic suppressors more GFP::RAB-3 is present in synaptic regions with lower accumulations in the cell body (Figure S2D). Consistent with this increase of GFP::RAB-3 in synaptic regions, the anterograde flux of GFP::RAB-3 in mechanosensory neurons is higher in the intragenic suppressors than in *unc-104(e1265)* (Videos S2, S3), but still significantly less than in wild type (Figure 4D, 4E, Video S1). There is a reduction in the anterograde velocity of GFP::RAB-3 only in *unc-104(e1265)* animals (Figure 4F), suggesting that in the suppressors, the partially functional UNC-104 motors that succeed in binding cargo are able to transport it efficiently. The retrograde flux of GFP::RAB-3 is also reduced in all three mutants, greatly so in *unc-104(e1265)* but only moderately in the suppressors (Figure 4D). The retrograde velocity is unaffected in all mutants (Figure 4F), suggesting that the reduced retrograde flux is likely due to fewer cargo vesicles being available for retrograde transport as a result of reduced anterograde transport.

Thus both intragenic suppressors that map to the PH domain improve behaviour and cholinergic synaptic transmission by increasing the transport of cargo in the axon and the number of synaptic vesicles at the synapse.

### Both intragenic suppressors partially restore preferential PI(4,5)P_2_ and cargo binding

To determine if the intragenic suppressors improve synaptic vesicle transport through improved cargo binding, we carried out a parallel analysis of the suppressors in a manner similar to *unc-104(e1265)*. The R1501Q mutation in *unc-104(e1265tb107)* is also on the surface of the PH domain (Figure 3A). It lies ∼7 Å away from D1497N within the PI(4,5)P_2_ binding pocket. The R1501Q may reverse the loss of charge in the PH domain variant encoded by *unc-104(e1265)* through compensatory local short range interactions. The compensatory change M1540I in the *unc-104(e1265tb120)* suppressor lies ∼20 Å from D1497N and is likely to mediate any possible effect on PI(4,5)P_2_ through long-range interactions (Figure 3A).

*In vitro* lipid binding using the D1497 R1501Q and D1497N M1540I PH domains showed that they partially restore preferential PI(4,5)P_2_ binding (Figure 3B, 3C). D1497N R1501Q shows a small increase in PI(4)P binding but D1497N M1540I does not show a similar increase. These suppressor variants of the UNC-104 PH domain, like the D1497N variant, continue to bind PC and PI at higher levels than the PH domain encoded by wild type (Figure 3B). These data suggest that along with partial restoration of preferential binding to PI(4,5)P_2_, some non-specific binding to lipids is still retained by the PH domains encoded by the intragenic suppressors.

As a confirmation that the intragenic suppressors are able to recognize PI(4,5)P_2_ *in vivo,* we observed that increased PI(4,5)P_2_ resulting from neuronal over-expression of *ppk-1* [35] leads in each suppressor to improved locomotion and reduced resistance to aldicarb induced paralysis (Figure 3D, 3E). This indicates increased transport to synapses resulting in increased vesicle release in the intragenic suppressors over-expressing PI(4,5)P_2_. The previously described [12] engineered mutants KK1463/4AA and R1496A are also similarly sensitive to PI(4,5)P_2_ levels *in vivo* (Figure S3E), suggesting that they do not reduce PI(4,5)P_2_ binding as severely as the D1497N variant.

Consistent with the *in vitro* and *in vivo* data, we observe that pre-synaptic vesicles prepared from *unc-104(e1265tb120)* animals have larger amounts of UNC-104 present on them than those prepared from *unc-104(e1265)* animals (Figure 2E). Both genotypes have nearly identical levels of synaptic vesicles as assessed by levels of the synaptic vesicle marker synaptobrevin (Figure 2E). Thus, compared to *unc-104(e1265)*, the proteins encoded by the intragenic suppressors (1) partially restore preferential PI(4,5)P_2_ binding *in vitro*, (2) are sensitive to PI(4,5)P_2_ levels *in vivo* (3) have more UNC-104 molecules on pre-synaptic vesicles and (4) facilitate transport of synaptic vesicles to synapses through an improved ability to bind cargo vesicles, leading to improved behaviour.

### Lack of binding to cargo results in loss of the UNC-104 motor, which is partially restored by both intragenic suppressors

To investigate consequences of cargo binding ability on the motor we examined the levels of the pan-neurally expressed UNC-104 motor in several alleles (Figure S4D). Greatest levels of endogenous UNC-104 are found in the synapse rich regions of the nerve ring and of the ventral cord (Figure S4A:a, Figure 4B). Lower levels of UNC-104 are present in the dorsal cord, sub-lateral cords and in neuronal commissural processes (Figure S4B). UNC-104 levels in *unc-104(e1265)* are greatly reduced compared to wild type animals and residual protein is still localized in the synapse rich regions of the nerve ring and ventral cord (Figure 4B, 4C:b, g, Figure S4A: c). As a comparison no change was observed in the levels or localization of the neuronal plasma membrane t-snare syntaxin (Figure 4C: k, l). In another pre-existing allele *unc-104(rh43)*, which encodes the motor UNC-104(G96E G314E) with a mutation in the ATP binding pocket of the motor domain (Figure S1A), the UNC-104 levels appear similar to wild type, although altered in distribution with significant increases in neuronal cell bodies (Figure 4C: c, h, Figure S4A: b). The altered distribution may arise from a motor that is unable to hydrolyze ATP and thus cannot walk efficiently along microtubules.

To see how partial restoration of the pattern that favours PI(4,5)P_2_ binding affects UNC-104 levels, we carried out immunohistochemistry and Western blots on both intragenic suppressors. We observed that the UNC-104 protein levels are also partially restored in the intragenic suppressors (Figure 4B, 4C: d,e,i,j). Moreover, this increase occurs where the endogenous levels of UNC-104 were highest, namely in the synapse rich regions of the nerve ring and of the ventral cord (Figure 4C: d,e,i,j). These regions also contain axons, so some of the increase could be taking place in axons. Upon increasing the *in vivo* levels of PI(4,5)P_2_ in intragenic suppressors, along with improved behaviour (Figure 3D, 3E), we see a further increase in UNC-104 levels (Figure S3F). Again this additional increase in UNC-104 levels is detected only in the synapse rich regions of the ventral cord (Figure S4I). This is likely due to an increased number of partially functional motors being recruited to cargo vesicles. (The above data are summarized in Table 1)

**Table 1:** Summary of all UNC-104 alleles and transgenes and their behaviour in multiple assays.

Allele	Transgene	*In vitro* PIP_2_ binding	*In vivo* PIP_2_ sensitivity	*In vivo* motor on synaptic vesicles	Rescue of *unc-104* function	UNC-104 Protein levels and localization
Wild type		High	Insensitive (saturated?)	High	----	High, largely in synapse-rich regions
*unc-104(e1265)* encodes UNC-104(D1497N)		Poor	insensitive	Very low	----	Very low, residual in synapse-rich regions
*unc-104(e1265tb107)* encodes UNC-104(D1497N R1501Q)		Medium	sensitive	More than in *unc-104(e1265)*	----	Medium, residual in synapse-rich
*unc-104(e1265tb120)* encodes UNC-104(D1497N M1540I)		Medium	sensitive	More than in *unc-104(e1265)*	----	Medium, residual in synapse-rich
	UNC-104::GFP	High	------	------	Full	Similar to wild type
	UNC-104(D1497N)::GFP	Poor	------	------	No	No GFP tagged protein observed
	UNC-104(D1497N)::GFP very high copy number	------	------	------	No	Similar to wild type
	UNC-104(M1540I)::GFP	------	------	------	Full	Similar to wild type
	UNC-104(D1497N M1540I)::GFP	Medium	------	------	Nearly full	Similar to wild type
	UNC-104(D1497N R1501Q)::GFP	Medium	------	------	Nearly full	Similar to wild type
	UNC-104(R1501Q)::GFP	------	------	------	Full	Similar to wild type
	UNC-104(W1549A)::GFP	------	------	------	Full	Similar to wild type
	UNC-104(D1497N M1540I W1549A)::GFP	Poor	------	------	Poor	No GFP tagged protein observed
	UNC-104(KK1463/4AA)::GFP high expression	Poor [12]	sensitive	-------	Poorer than UNC-104(D1497N M1540I)::GFP	Similar to wild type
	UNC-104(R1496A)::GFP high expression	Poor [12]	sensitive	-------	Poorer than UNC-104(D1497N M1540I)::GFP	Similar to wild type

Taken together, our observations show that the *in vivo* levels of the UNC-104 motor are directly related to its ability to bind pre-synaptic vesicles through PI(4,5)P_2_, suggesting a link between specific binding of a motor to its cargo and levels of the motor in neurons.

### UNC-104 motors with reduced cargo binding ability are degraded, at least partly through the ubiquitin pathway

We wished to test if the reduced UNC-104 levels in the *unc-104* variants are due to its degradation. To rule out reduction in transcripts, we measured RNA levels of UNC-104 using real-time PCR. We saw no change in UNC-104 RNA levels between wild type and *unc-104(e1265)* animals (Figure S3D). To study other possible effects of the D1497N mutation on the UNC-104 motor such as altered localization or motility, we compared transgenic animals expressing high levels of UNC-104::GFP and UNC-104(D1497N)::GFP. High levels were used since at low levels, there is almost no expression of the mutant motor *in vivo*. Both variants show similar localization and nearly identical microscopic movements (Figure 4G, see below). Nearly 85% of UNC-104::GFP and 75% of UNC-104(D1497N)::GFP molecules that move do so in the anterograde direction while approximately 15-25% move in the retrograde direction (Figure 4G). Thus mis-localization or immobility of the UNC-104 motor are unlikely to underlie the observed phenotypes of *unc-104(e1265)* animals.

**Figure 5:**
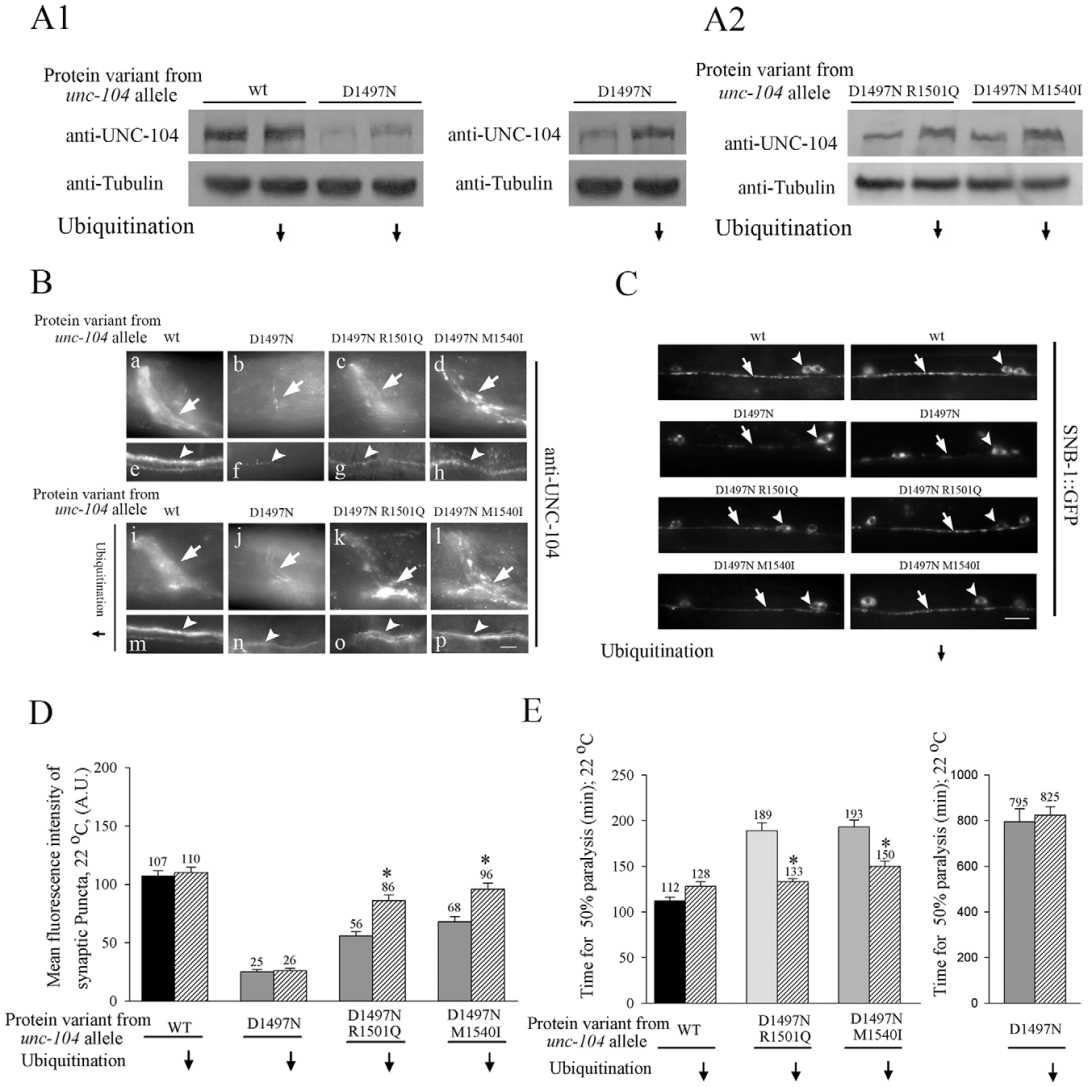
Levels of UNC-104 motor in cargo binding variant alleles depends on ubiquitination. Ubiquitination is reduced using the mutant *uba-1(it129ts)* at 22°C. (A1,A2) Anti-UNC-104 western blots. (A1) Total UNC-104 levels increase in *unc-104(e1265)* upon reducing ubiquitination. The same western blot with two different exposures has been provided for *unc-104(e1265)*. (A2) UNC-104 levels increase in *unc-104(e1265tb107)* and in *unc-104(e1265tb120)* upon reducing ubiquitination. (B) Anti-UNC-104 immunoreactivity in all *unc-104* cargo-binding variants in *uba-1(it129ts)* background. Upon reducing ubiquitination, UNC-104 levels increase in *unc-104(e1265)*, *unc-104(e1265tb107)* and *unc-104(e1265tb120)* in synapse rich regions of the nerve ring (arrow) and ventral cord (arrowhead). (C) Localization of SNB-1::GFP cargo markers in neuromuscular junctions observed using *juIs1* in *unc-104* cargo binding variant alleles. Upon reducing ubiquitination, cargo marked by synaptobrevin increases at synapses in *unc-104(e1265tb107)* and in *unc-104(e1265tb120)*. Arrowhead points to ventral cord motor neuron synaptic puncta. Arrow points to the cell body. (D) Mean fluorescence intensity of neuromuscular junction synaptic puncta marked by synaptobrevin::GFP. The intensity of synaptic puncta in *unc-104(e1265tb107)* and *unc-104(e1265tb120)* increases upon blocking ubiquitination. (n = 10 animals and *p<0.05). Data represented as mean ± SEM. (E) Aldicarb resistance assay in the UNC-104 PH domain variant alleles. Time for aldicarb induced paralysis decreases in *unc-104(e1265tb107)* and in *unc-104(e1265tb120)* upon block in ubiquitination. n = 30 animals, done three times independently. Data represented as mean ± SEM (*p = 0.005). The alleles *unc-104(e1265)*, *unc-104(e1265tb107)* and *unc-104(e1265tb120)* are labeled in the figure by the respective protein changes they encode, namely D1497N, D1497N R1501Q and D1497N M1540I.

To test if UNC-104 is degraded in the *unc-104* allelic variants we built double mutants between these variants and the temperature sensitive allele of the E1 Ubiquitin ligase *uba-1(it129ts)* [26]. We observed a small but consistent increase in UNC-104 levels on Western blots in *unc-104(e1265); uba-1* animals grown at 22°C compared to *unc-104(e1265)* animals grown at the same temperature (Figure 5A1). This increase, observed primarily in the nerve ring (Figure 5B: b, f, j, n), did not result in any improvement in resistance to aldicarb (Figure 5E), probably because the mutant motors are still unable to bind cargo efficiently for transport. Similar results were obtained for the two intragenic suppressors. Western blots showed increased UNC-104 levels in each suppressor in the *uba-1* background (Figure 5A2). Again this increase occurs in the synapse rich regions of the nerve ring and of the ventral cord (Figure 5B: c,g,k,o,d,h,l,p). These regions also contain axons, so the increase in motors may occur to some degree in axons in addition to synapses.

Concomitant with the increase in motor levels, we observed a significant increase in synaptic vesicles at neuromuscular junction synapses in *unc-104(e1265tb107)* and *unc-104(e1265tb120)* in the *uba-1* background (Figure 5C, 5D). This was reflected in better behaviour, namely we saw greater sensitivity to aldicarb at 22°C in both suppressors in the *uba-1* background (Figure 5E). These data indicate that blocking the ubiquitin-mediated degradation pathway in the suppressors increases the numbers of partially functional motors, which likely improves the transport of pre-synaptic vesicles, resulting in improved synaptic transmission.

Taken together, our observations suggest that loss of ability to bind cargo can lead to motor degradation in neurons. Further, the UNC-104 motors that have reduced binding ability to PI(4,5)P_2_ are degraded at least partially through the ubiquitin pathway in synapse rich regions of the nerve ring and ventral cord.

### Transgenic variants, except UNC-104(D1497N), restore function and motor protein levels

**Figure 6:**
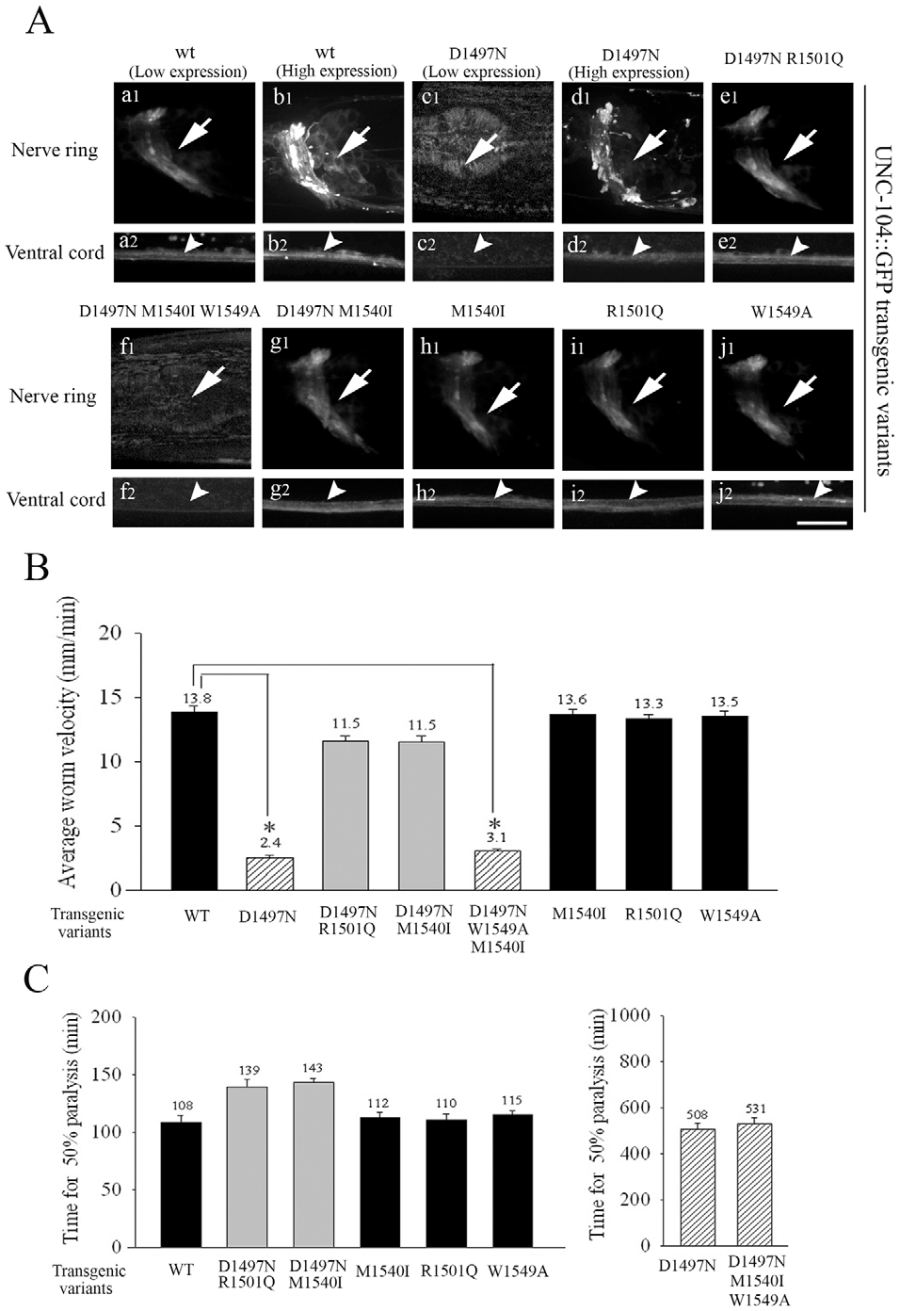
Localization and behavioural rescue of UNC-104::GFP transgenes with different PH domain mutations. (A) Expression and localization of UNC-104::GFP PH domain variants in *unc-104(e1265)* background. All transgenic animals except those with the D1497N and D1497N M1540I W1549A mutations in the PH domain express GFP at high levels. Over-expression of UNC-104(D1497N)::GFP shows expression and localization of the protein similar to high copy number UNC-104::GFP transgenic animals. In all transgenes that show expression, the localization of UNC-104 is similar to wild type UNC-104::GFP. Scale bar: 10 µm. (B) Locomotory behaviour of *unc-104(e1265)* animals carrying various UNC-104::GFP transgenes. All transgenes except UNC-104(D1497N)::GFP (*p = 10^-31^) and UNC-104(D1497N M1540I W1549A)::GFP (*p = 10^-29^) rescue locomotry behaviour. While UNC-104(D1497N R1501Q)::GFP and UNC-104(D1497N M1540I)::GFP also provide rescue, they are significantly different from wild type (*p = 10^-7^, 10^-6^). UNC-104(D1497N) does rescue viability of the *unc-104(rh142)*, a null allele (data not shown). All data represented as mean ± SEM. (C) Aldicarb resistance of *unc-104(e1265)* animals carrying various UNC-104::GFP transgenes. UNC-104(D1497N)::GFP (*p = 0.001) and UNC-104(D1497N M1540I W1549A)::GFP (*p = 0.007) animals are highly resistant to aldicarb and do not provide functional rescue. All other transgenes confer aldicarb resistance similar to wild type and provide functional rescue. All data represented as mean ± SEM.

To provide further support for the observed loss of the UNC-104 motor upon lack of PI(4,5)P_2_ binding, we made low copy number transgenic lines of several UNC-104 variants by bombardment into *unc-104(e1265)* animals. The UNC-104 motor variant transgenic lines were made using wild type UNC-104, UNC-104(D1497N), UNC-104(D1497N R1501Q), UNC-104(D1497N M1540I) with and without a GFP fused to the C-terminus expressed under the control of the *unc-104* promoter. The GFP containing and GFP lacking transgenic lines behaved identically in both locomotion and aldicarb resistance assays, suggesting that addition of GFP did not alter the function of the UNC-104 variants (Figure S3B1, S3B2, S3C1, S3C2). All variants except UNC-104(D1497N) provided full or partial rescue of the localization of GFP::RAB-3 in a pattern similar to that observed in wild type animals (Figure S4E). All transgenic lines except UNC-104(D1497N)::GFP provide significant restoration of both locomotion and synaptic transmission as assayed by aldicarb sensitivity (Figure 6B, 6C). All transgenic lines except UNC-104(D1497N)::GFP express GFP in a pattern similar to the pattern of immunoreactivity seen in wild type animals (Figure 6A). However none of the three independently generated UNC-104(D1497N)::GFP transgenic lines express GFP (Figure 6A: c1,c2). Further, injecting the UNC-104(D1497N)::GFP construct at high DNA concentrations (∼200ng/µl) did result in motor-GFP expression in a pattern similar to high copy number UNC-104::GFP transgenic lines (Figure 6A: b1,b2,d1,d2; Figure S4C). We think that this expression in very high copy number transgenic UNC-104(D1497N)::GFP lines is likely due to saturation of the endogenous degradation machinery. These transgenic animals clearly demonstrate that some fusion protein expressing GFP could be produced by this construct when sufficiently high copy numbers of the encoding DNA are provided but this expression still does not provide behavioural rescue (data not shown, all transgenic data are summarized in Table 1). However when expressed at levels closer to endogenous levels, the UNC-104(D1497N) variant is not detectable, possibly due to being targeted for degradation. The most parsimonious conclusion is that specific binding to PI(4,5)P_2_ molecules present on cargo vesicles is essential for maintaining the levels of the UNC-104 motor.

### Mutating the conserved W1549 in UNC-104(D1497N M1540I) abolishes preferential PI(4,5)P_2_ binding, leading to loss of motor

To further confirm that the ability to maintain preferential PI(4,5)P_2_ binding is co-related to *in vivo* motor levels, we mutated the W1549 to A. The intragenic suppressor M1540I carries out its suppression indirectly. This residue is ∼2.4 Å away from the highly conserved Trptophan at 1549. In the homology model, I1540 orients its β carbon methyl group towards W1549, which in turn lies close to KK1463/4 (∼7.8 Å/3.2 Å respectively) (Figure 3A). We predicted that the W1549 residue would mediate the suppression of M1540I through interaction with the classical KK1463/4 residues. KK1463/4 are known to play important roles *in vitro* in binding PI(4,5)P_2_ [12]. Since the side chain of isoleucine is bulkier than methionine, M1540I mutation might be amenable for better interaction with the conserved W1549. This may directly cause a change in the binding site for better presentation to the ligand. Thus, changing the W1549 to A1549 is likely to reduce the presumptive interaction from I1540 to the KK1463/4.

Therefore, we tested the *in vitro* lipid binding specificity of an UNC-104 PH domain carrying (D1497N M1540I W1549A) mutations and observed that this triple mutation abolishes the preferential PI(4,5)P_2_ binding *in vitro* while increasing binding to PC and PI, and behaves similarly to the D1497N mutation alone (Figure 3B, 3C). As predicted, this triple mutation abrogates the ability of the M1540I to suppress the deleterious effects of the D1497N lesion.

We also made low copy number integrated transgenic lines using bombardment with both UNC-104(W1549A)::GFP and UNC-104(D1497N M1540I W1549A)::GFP into *unc-104(e1265)*. The UNC-104(W1549A)::GFP lines exhibit wild type locomotory behaviour, sensitivity to aldicarb and motor expression levels and localization (Figure 6A: j1,j2), suggesting that a motor with W1549A does not materially alter function *in vivo* (Figure 6B, 6C, Figure S3B1, S3B2). However the UNC-104(D1497N M1540I W1549A)::GFP does not show any expression of GFP in any of the transgenic lines generated (Figure 6A:f1,f2). Nor does it exhibit normal locomotion and moreover the *unc-104(e1265)* animals carrying this transgene continue to be resistant to aldicarb (Figure 6B, 6C). Thus all analyzed mutations that reduced the preferential PI(4,5)P_2_ binding also show reduced UNC-104 motor protein levels.

## Discussion

We provide the first evidence that the *C. elegans* UNC-104/KIF1A motor does not return from the synapse, is degraded *in vivo* through the ubiquitin pathway and that the degradation takes place in synaptic regions. To study a possible mechanism underlying this degradation, we developed an allelic series of mutants that either strongly or moderately affect the ability of the UNC-104 motor to bind PI(4,5)P_2_ and hence pre-synaptic vesicles, leading to corresponding failure of pre-synaptic vesicles to reach synaptic regions. In these mutants, levels of the UNC-104 motor depend on its ability to bind cargo and moreover the motor is degraded in synapse rich regions of the nervous system. These data together provide support to the hypothesis that the UNC-104 motor is degraded at synaptic regions upon releasing its synaptic vesicle cargo.

### Directionality of anterograde transport

Failure of UNC-104 to return from synaptic regions in *C. elegans* neurons (Figure 2 A-C) corroborates prior reports showing, via axon ligation assays, that motors such as the mammalian KIF1A, Kinesin-1 and KIF3A/B do not get retrogradely transported back to the cell body [6], [21], [22]. Degradation at synapses can explain this apparent macroscopic directionality of anterograde transport. Such degradation could have consequences for cargo transport, for instance, by providing a mechanism for preventing tug-of-war with a retrograde motor or return of retrogradely directed cargo back to the synapse.

### Possible mechanism for motor degradation

That UNC-104 is degraded near synaptic regions is demonstrated by increase in motor levels at synapses in mechanosensory neurons upon blocking ubiquitin-mediated degradation (Figure 1A:e1,e4). Together with the observed *in vivo* ubiquitination of UNC-104 (Figure 2D), this suggests that degradation of the motor at synapses is mediated directly or indirectly by ubiquitination. The degradation of UNC-104 through the ubiquitin pathway is likely to require the PH domain since a transgenic motor::GFP fusion protein lacking the PH domain has been shown to be highly expressed [12] (Figure S4C). This explanation is also consistent with the observed direct interaction of ubiquitin with a split PH domain that shares significant homology to the UNC-104 PH domain [39]. Further, the UNC-104 PH domain has 70% similarity to a 43 amino acid ubiquitin-mediated degradation sequence found in kinesin Kip1p [40]. Moreover several lysine residues are present in the PH domain, including three in the PH domain that may be targets for attaching ubiquitin to the UNC-104 motor (Figure S1B). While these facts suggest that the degradation of UNC-104 is likely to occur via ubiquitin interactions with the PH domain of the motor, we cannot rule out other degradation pathways, for instance involving a more indirect role for ubiquitination and/or a role for phosphoinositides [41].

### Observed characteristics in the allelic series of UNC-104 mutants

In the allelic series consisting of wild type, *unc-104(e1265)* and its two intragenic suppressors, the ability to bind PI(4,5)P_2_ determines the levels of motors on pre-synaptic vesicles *in vivo*, the extent of transport of synaptic vesicle proteins to the synapse, and hence the extent of locomotion and of synaptic transmission. We think that the primary defect in these *unc-104* mutants is differential abrogation of cargo binding ability, rather than other effects such as altered localization, motility, folding or stability of the mutant motor. The fact that over-expressed UNC-104(D1497N)::GFP and over-expressed UNC-104::GFP localize and move similarly *in vivo* (Figure 6A, Figure 4G) argues against localization and motility being affected. We cannot currently exclude the possibility that protein folding or stability is changed *in vivo*. We discuss this in the next section.

We also found the levels of UNC-104 in all alleles to be directly related to their cargo binding ability. Further the mutant motors undergo ubiquitin-mediated degradation in synapse rich regions of the animal, as seen by the small increase (see the next paragraph) in UNC-104 expression in these regions after blocking ubiquitin-mediated degradation (Figure 5B). The D1497N lesion in itself is unlikely to cause the mutant UNC-104 motor to be targeted for degradation since the D1497N residue is not a direct target for ubiquitin conjugation, and hence UNC-104(D1497N) is unlikely to generate a new site for poly-ubiquitin attachment.

The likely reason why only a small increase is observed in mutant UNC-104 levels upon blocking ubiquitination is that *uba-1* is a mild temperature sensitive mutant providing sufficient function for viability of the *uba-1* animals. This is also the probable reason behind the apparent lack of change seen in endogenous UNC-104 levels in *uba-1* mutants alone in these assays (Figure 5A1, 5B: i,m). One would expect to see such a change in view of the independently established degradation of the UNC-104 motor in mechanosensory neurons (Figure 1A:e1,e4). But since endogenous wild type UNC-104 is present in all neurons in large amounts, we think that the small change caused by *uba-1* is difficult to detect. It may be possible to see more robust effects, including on endogenous wild type UNC-104, if one identifies a specific E3 ubiquitin ligase, rather than using a general block of degradation provided by *uba-1.*

### A proposed relationship between cargo release and UNC-104 degradation

The observed degradation patterns of UNC-104 in the animals in the allelic series, coupled with the direct relationship between UNC-104 levels in these animals and cargo binding ability of the mutant motors, provide support to the following hypothesis. The endogenous UNC-104 motor carrying synaptic vesicles goes to synaptic regions and is degraded there upon cargo release.

At present we cannot rule out potential instability of the mutant UNC-104 motor as the primary factor leading to its degradation and hence to loss of cargo binding and other ensuing phenotypes. However this explanation is considerably less parsimonious since it leaves unexplained the following localization and movement patterns of mutant motors. Observed steady state localization of all three mutant motors is confined to synapse rich regions, as is the increase upon blocking ubiquitination (Figure 5B). This suggests that the mutant motors can get transported to synaptic regions. Further, the microscopic movements of over-expressed UNC-104(D1497N)::GFP suggests that at least some mutant motors are able to fold and move correctly (Figure 4G). Moreover, the nearly identical CD melting spectra of both the wild type and D1497N PH domains imply their structural similarity (data not shown).

The fate of UNC-104 motors not carrying synaptic vesicles is less clear. In case of the mammalian KIF1A, motors unbound to cargo have recently been shown to be held in an auto-inhibited state preventing transport to neurite tips [17]. However there are reported differences between UNC-104 and KIF1A, e.g., UNC-104 appears to exist as a monomer and is thought to dimerize on the surface of the cargo [14], whereas KIF1A has been reported to move as a monomer [42] and recently it is reported to be held as a dimer [17]. Moreover UNC-104 has been previously reported to enter axons even after deletion of its cargo binding PH domain, demonstrating that cargo binding is not necessary for movement of the motor (Figure S4C) [12]. In all our allelic variants including wild type, we find almost no UNC-104 present in most neuronal cell bodies, even in the *uba-1* background (Figure 5B: e, m, o, p contrast with Figure 4C: h). One possible explanation is that most motors enter the axon very quickly – with mutant versions conceivably carrying other lipids or even no cargo – and upon reaching synaptic regions and after losing binding to cargo, the motor is rapidly targeted for degradation. Other mechanisms, such as degradation of motor as soon as the motor-cargo complex reaches the synapse or only after inactivation of the motor, cannot be excluded. However, our work suggests that a plausible mechanism is one in which release of the motor from its cargo may expose free motors to degradation at or near the synapse.

## Materials and Methods

### Modeling of UNC-104 PH domain

A BLAST search of the UNC-104 PH domain sequence against the RSCB protein data bank identifies DAPP1/PHISH (Dual adaptor of phosphotyrosine and 3-phosphoinositides, from *Homo sapiens*, PDB code 1FB8) as the closest homolog. The two sequences were then aligned using CLUSTAL W (EBI server) and carefully adjusted using manual intervention, to ensure maximum conservation of motifs and minimal gap regions. A homology model was then generated using MODELLER v7.0 [43]. Output structure was relaxed with 500 steps of energy minimization (Steepest Descent) using SYBYL (Tripos Associates, Inc.). The energy-minimized structure was then used as input for docking PI(4,5)P_2_ using GRAMM [34].

### Bacterial protein expression and purification for *in vitro* lipid binding assays

The starting constructs for all was an UNC-104 PH domain fused in frame to GFP [25]. Various point mutations (D1497N, D1497N M1540I, D1497N R1501Q, D1497N W1549A M1540I) in the PH domain were generated using site directed mutagenesis using the Stratagene QuickChange protocol with *TaKaRa Ex Taq*. PH domain constructs were cloned into pET17b vector and all constructs were verified by DNA sequencing. The proteins were expressed in Rosetta bacterial cells (Invitrogen), purified by Ni-NTA chromatography (QIAGEN) and kept frozen in 10mM Tris pH 8.0, 4mM EGTA, 5% sucrose.

### Liposome preparation

The followings lipids were purchased from Avanti Polar lipids. Egg PC (Cat. no. 840051), PI(4)P (Cat. no. 840045), PI(4,5)P_2_ (Cat. no. 840046), PA (Cat. no. 840101), PI (Cat. no. 840044) and BL (Cat. no. 131101). Composition of brain lipids (BL) contains Phosphatidylethanolamine (16.7%), Phosphatidylserine (10.6%), Phosphatidylcholine (9.6%), Phosphatidic acid (2.8%), Phosphatidylinositol (1.6%) and others (58.7%).

5 µM concentration of the desired lipids was used to prepare liposomes in the following ratio: 10% desired lipid and 90% carrier lipids. Phosphatidylcholine (PC) was used as a carrier lipid and the remaining 10% of the lipids used were either Phosphatidylinositol (PI), Phosphatidylinositol-4-phosphate PI(4)P, Phosphatidylinositol-4,5-bisphosphate PI(4,5)P_2_ or brain lipid (BL) in chloroform. After mixing the desired and carrier lipids, chloroform was evaporated under a constant Nitrogen gas stream. Once the lipid film was dried completely, lipids were rehydrated by the addition of LB buffer (30 mM tris, 4 mM EGTA, pH 8.0). These lipids were sonicated (ultrasonic bath) for 30 seconds to break up the lipid aggregates and were then extruded through a 100 nm pore polycarbonate filter (Avestin, Ottawa, Canada) using a miniextruder from Avanti polar lipids. The liposomes were stored in the dark at 4°C and used within a week of preparation.

### Liposome binding assay

Liposomes were prepared as previously described [14]. Briefly, liposomes (5 µM total lipid concentration) were prepared in LB buffer (30 mM tris, 4 mM EGTA, pH 8.0). 100 µl of freshly prepared liposomes were mixed with about 1 µg protein and incubated on ice for 30 min. The incubation reaction mixtures were centrifuged at 50000g_av_ (4°C) for 45 min in a TLS-120 rotor (Beckman). After centrifugation, fractions from the pellet that contains liposome bound protein and supernatant that contains unbound protein were collected. Samples were dissolved in 20 µl LB buffer and analyzed by SDS-PAGE followed by Coomassie staining. Gels were digitized on a flatbed scanner and protein bands were quantified using ImageJ (version 1.37, NIH). Binding specificity was determined by normalizing binding observed with PI(4,5)P_2_ and brain lipid compared to binding observed using PC alone carrier liposomes.

### *unc-104(e1265)* suppressor screen

L4 *unc-104(e1265)* worms were washed with M9 buffer, using sterile glass pipettes. Washed worms were transferred into a tube of 1x PBS containing ethyl methanesulfonate (Sigma) at a final concentration of 50mM. Tubes were kept in a rotary shaker at 20°C for 4 hours. After mutagenesis, 3-4 worms were transferred each 60 mm Petri plate. F1 and F2 progeny were regularly examined under a Nikon SMZ645 dissecting microscope for improved locomotion in a non-clonal screen of approximately 60,000 haploid genomes. Intragenic suppressors were identified in genetic crosses that mapped them close to the *unc-104* locus. Intragenic suppressors of *unc-104(e1265)* isolated were *unc-104(e1265tb107)* and *unc-104(e1265tb120)*. Throughout the paper, proteins encoded by these alleles are referred to as UNC-104(D1497N), UNC-104(D1497N R1501Q) and UNC-104(D1497N M1540I) respectively.

### Worm motility assays

1 day adult hermaphrodites were transferred on to a fresh NGM agar plate, allowing them to acclimatize for 1 hour. Movement was recorded on a Nikon SMZ800 dissecting microscope at 1 to 1.3 frames per sec (1000×1000 pixels) for 2-3 min with a cooled monochrome camera (Evolution Qei, Media Cybernetics). Movement was tracked manually using ImageJ (version 1.37, NIH) software. Worms that moved for a minimum of 10 frames were tracked. Worm velocities were obtained by calculating the straight line distance between the centroid positions of the worm in a given interval.

### Aldicarb assays

Aldicarb plates were prepared by adding aldicarb (Chemical Service, Westchester, PA) solution (in 70% ethanol) to NGM agar. These plates were seeded with OP50 bacteria. All assays were performed on 1 day old adult hermaphrodites at room temperature (21-23°C). 30 individuals were incubated for 6-8 hr on aldicarb plates of defined concentration. At 30 min intervals each worm was touched with a platinum wire and was checked for paralysis [36]. Aldicarb inhibits acetylcholine esterase causing the neurotransmitter acetylcholine to persist longer at the synapse and hyperstimulate the post-synaptic sites. This leads to loss of co-ordinated motion and finally paralysis. Faster paralysis indicates more acetylcholine release at synapses. In our experimental context wild type paralyzes the fastest while mutants that do not have vesicles to release paralyze the slowest. Any reduction in paralysis time indicates more vesicles present at synapses for release.

### Constructs

A wild type UNC-104::GFP construct was provided by Jon Scholey [25]. This construct harbours the *unc-104* promoter driving the combination of intronless and genomic region of *unc-104* and provides the entire open reading frame of the protein. Mutations were introduced using site directed mutagenesis using the Stratagene QuickChange protocol with *TaKaRa Ex Taq*. Various point mutations (D1497N; D1497N M1540I, M1540I, D1497N R1501Q, R1501Q, W1549A, D1497N M1540 W1549A) were generated. All constructs were verified by DNA sequencing. GFP was deleted from UNC-104::GFP, UNC-104::GFP(D1497N), UNC-104::GFP(D1497N R1501Q) and UNC-104::GFP(D1497N M1540I) using the restriction enzymes Apa1 and Kpn1. After T4 DNA polymerase treatment, ligation was done using T4 DNA ligase.

### *C. elegans* strains

Worms were grown at 20°C on NGM agar plates seeded with *E.coli* Strain OP50 under standard laboratory conditions (Brenner, 1974). Strains used in the study, provided by the Caenorhabditis Genetics Center (CGC), are as follows: wild type *N2, unc-104(e1265), unc-104(rh43), unc-104(rh142).*

*juIs1*(*_p_unc-25-SNB-1::GFP*) a transgenic strain expressing green fluorescent protein (GFP)-tagged synaptobrevin-1 in GABA motor neurons [24], [37]

*jsIs1 (_p_snb-1::snb-1::GFP)* a transgenic strain that expresses SNB-1::GFP in all neurons [44].

*zdIs5*(*_p_mec4::GFP*) a transgeneic strain expressing soluble GFP in mechanosensory neurons [29]

*jsIs821*(*_p_mec7::GFP::RAB-3*) a transgenic strain expressing GFP tagged RAB-3 in mechanosensory neurons [27]

*trIs25 (him-4p::MB::YFP, F25B3.3P::DsRed2)* has Membrane–anchored yellow-fluorescent protein expressed in body wall muscles [38].

*jsIs682(_p_rab-3::gfp::rab-3)* a transgenic strain that expresses GFP::RAB-3 pan-neurally [27].

*gqIs125(_p_rab-3::ppk-1*) a transgenic strain that over-expresses the PI(4,5)P_2_ biosynthetic enzyme Type I PIP kinase *ppk-1* in all neurons [35]

*uba-1(it129ts)* is a temperature sensitive mutant allele in the E1 ubiquitin activating enzyme [26]

*js1111 (_p_mec4::UNC-104::GFP)* a transgenic strain that expresses UNC-104::GFP only in mechanosensory neurons.

### UNC-104 transgenes are all expressed pan-neurally under its endogenous promoter

UNC-104- 5 transgenic lines- *tbIs183*

UNC-104::GFP- 5 transgenic lines- *tbIs147*

UNC-104(D1497N)- 3 transgenic lines- *tbIs188.* This transgene rescues *unc-104(rh142)*, the lethal null allele

UNC-104(D1497N)::GFP- 3 transgenic lines- *tbIs149*. This transgene provides viability to *unc-104(rh142)*, the lethal null allele

UNC-104(D1497N R1501Q) - 3 transgenic lines- *tbIs194*

UNC-104(D1497N R1501Q)::GFP- 3 transgenic lines- *tbIs152*

UNC-104(D1497N M1501I)- 4 transgenic lines- *tbIs191*

UNC-104(D1497N M1501I)::GFP- 3 transgenic lines- *tbIs156*

UNC-104(M1540I)::GFP-13 transgenic lines- *tbIs157*

UNC-104(R1501Q)::GFP- 4 transgenic lines- *tbIs170*

UNC-104(D1497N M1540I W1549A)::GFP- 5 transgenic lines- *tbIs199*

UNC-104(W1549A)::GFP- 2 transgenic lines – *tbIs181*

Underlined strain was most commonly used, at least one other transgenic strain was assayed in all assays and no co-injection marker was used to make the above transgenic animals.

### Transgenic development

Micro particle bombardment of *C. elegans unc-104(e1265)* hermaphrodites was carried out using a BioRad Biolistic PDS-1000/HE particle delivery system (Bio-Rad Laboratories, Hercules, CA, USA) [45]. For each bombardment, 5-6 µg plasmid DNA was fixed to 0.5mg of 1.0 µm micro carrier tungsten particles, as described in the PDS-1000/HE user's manual, and bombarded on to a monolayer of unc*-104(e1265)* L4. Worms were allowed to recover for 0.5 to 1 hr after bombardment and were then transferred on to 100mm seeded Na22 plates and grown at 20°C. After 8-12 days worms were screened for improved movement and/or GFP expression as examined using a Zeiss fluorescence microscope. Individual animals were cloned. Homozygous stable lines were identified by the complete absence of *unc-104(e1265)* mutant progeny over several generations [45]. We used *unc-104(e1265)* as the background for bombardment since this was the healthiest hypomorphic allele of *unc-104* available.

### Image acquisition and analysis

For quantitation of SNB-1::GFP puncta at motor neuron synapses synaptic, unsaturated images of immobilized worms were taken in the linear range of exposure and quantified using ImageJ (NIH) similar to what has been described in [46].

For *in vivo* live imaging, young adult hermaphrodites were immobilized with 3-5mM levamisole (Sigma-Aldrich) in M9 and mounted on a 2% agarose pad. Time-lapse images of anterior mechanosensory neurons expressing GFP::RAB-3 were obtained with OLYMPUS IX81 using 100X/1.4 NA plain Apochromat objective attached with spinning disk confocal head (YOKOGAWA CSU22) equipped with EMCCD camera (ANDORiXon-897EMCCD). Time-lapse images (512×512 pixels) were taken at a constant frame rate of 6-7 frames per second. Image analysis was done using Image J (version 1.37, NIH). Kymographs were obtained from lines that were drawn along the axon from cell body towards synapse. Flux analysis was carried out within a range of 15-20 µm along the axon length, at a distance of 15-25 µm away from the cell body. Flux was calculated as number of anterogradely moving particles in a movie. Any particle static for 3 frames or with velocity less than 0.3 µm/s was considered as stationary. Pause frequency was calculated as the number of pauses taken by a particle for unit distance traveled (number of pauses/total distance traveled).

### Statistical analysis

All significance was calculated using pair-wise comparisons using the Student's T-test with unequal variance. p values less than 0.05 were considered as significant.

### Monoclonal antibody generation

The protein region of UNC-104 (amino acid 740-1117) was cloned into pRSETA vector (Invitrogen) using standard techniques. Protein was expressed in BL21 cells (Invitrogen), and purified using Ni-NTA chromatography (QIAGEN). Purified protein was given to Bioklone, Chennai, India to generate monoclonal antibodies. Specificity of the antibodies was tested by immunostaining *unc-104(rh142)*, a null allele. All monoclonal antibodies tested showed pan-neural staining in wild type animals and no staining in the *unc-104(rh142)* animals (Figure S4D).

### Immunostaining and western blots

Animals were fixed with 2% paraformaldehyde for 10 minutes at 4^o^C and freeze-thawed using liquid nitrogen and fixed for an additional 10 minutes at 4° C. Following this 4-5 washes with 0.5% BT buffer (20mM H_3_BO_3,_ 0.5% TritonX-100, pH 9.5) and then 5-6 washes (1 hour each) with 0.5%BTB (BT with 2% mercaptoethanol) were carried out. Blocking was done with PBST (phosphate buffered saline, 0.5%BSA, 0.5% TritonX-100, 10mM sodium azide). Samples were incubated two overnights with monoclonal anti-UNC-104 antibody (1:5), washed for 4-5 times with PBST (each of 15 minutes) before mounting. Rabbit anti-syntaxin was used at 1:10,000 [47]. Appropriate secondary antibody (1:200) incubations (anti-mouse Alexa 488, Alexa 568) were done for two overnights at 4° C. Images were captured using Zeiss Axiovert inverted microscope. Images were processed with Adobe Photoshop Version 9.0.

Western sample of worms were prepared by sonication. After sonication, worm lysates were boiled with SDS lysis buffer and proteins were separated on SDS PAGE (8% acrylamide). Proteins were transferred to a nitrocellulose membrane (Amersham), probed with a mouse serum or a mouse monoclonal antibody of anti-UNC-104 (1:60), rabbit anti-tubulin (1:1,000) (Thermo-scientific), rabbit anti-synaptobrevin (1:5000) [44] and rabbit anti-ubiquitin (1:500) (Sigma-Aldrich) followed by HRP based chemiluminescence detection (Pierce). Exposure time was varied from 30 seconds to 5 minutes, scanned and intensities quantitated using ImageJ. These intensities were pooled from multiple experiments and graphed and the exposure time chosen was determined to be in the linear range for all genotypes.

### FRAP experiments and analysis

Worms of respective genotypes were anesthesized in 5mM levamisole. Photobleaching experiments were done on confocal Zeiss LSM-5 Live (line scanner) equipped with a 63X objective (oil immersion, 1.4 NA) with a 488 nm solid state laser. Images were acquired on a CCD camera at the frame rate of 4 Hz. 35-40 µm of the axon was bleached across the synaptic branch. Fluorescence recovery was quantified from the distance covered by the UNC-104::GFP signal in bleached axons at fixed times after bleaching. The fluorescent recovery along the anterograde and retrograde directions was represented as velocity in both anterograde (recovery from cell body) and retrograde (recovery from synapse) directions. All the analysis was done using ImageJ version1.41 (NIH).

### Immunoprecipitation and sucrose gradient sedimentation

N2 worms were used for immunoprecipitation. For sedimentation assays we used *jsIs1* and various *unc-104* mutants in the *jsIs1* background. The worms and various mutants were grown on 10-15 large plates until food was exhausted. Worms were mechanically homogenized in homogenization buffer (15mM HEPES-NaOH pH 7.4, 10 mM KCl, 1.5 mM MgCl_2,_ 0.1 mM EDTA, 0.5 mM EGTA 0.05 M sucrose and protease inhibitors (Roche) and mildly sonicated at 4°C. The final supernatant was centrifuged at 50,000g for 40 min in a TLA 100.3 rotor to clear debris and heavy membrane fractions. The supernatant was collected again and centrifuged at 175,000g in TLA100.3 rotor for 150 min. The final pellet was resuspended in homogenization buffer or IP buffer (20 mM HEPES, 40 mM KCl, 5 mM EGTA, 0.1m M EDTA, 5 mM MgCl_2_ with protease inhibitors) as needed.

For immunoprecipitation the high speed re-suspended pellet was incubated with specific antibody for 5-6 hrs at 4°C. Final concentration of UNC-104 antibody used was 1:10 and ubiquitin antibody (Sigma-Aldrich) used was 1:10. Protein A agarose beads were added to the antigen-antibody mixture and incubated for 3-4 hours at 4°C. The beads were centrigufed, washed with IP buffer then analyzed by western blotting. A Western analysis was carried out on immunoprecipitated material using the anti-UNC-104 antibody and anti-ubiquitin antibody. The blot was first probed for UNC-104 and then stripped (no signal was observed after stripping) and re-probed for ubiquitin (1:500) (Sigma-Aldrich). The anti-ubiquitin antibody recognized the same band detected by anti-UNC-104. A Western analysis was carried out on immunoprecipitate obtained using the anti-ubiquitin antibody. This blot was probed using anti-UNC-104 and a band that migrates at the same size as endogenous UNC-104 was observed.

For sucrose gradient density, the resuspended high speed pellets were loaded on a discontinuous sucrose gradient centrifugation (0.05 M, 0.6 M, 1 M and 1.5 M) and centrifuged in a SW41 rotor at 60,000g for 120 min. Fractions were collected from top of the gradient up to the first layer (between 0.05M-0.6M). The last two fractions collected were below the formed layer where no synaptic vesicle proteins were detected. Western blot analysis with exposure maintained in the linear range was carried out on the fractions collected.

## Supporting Information

Figure S1 (A) A schematic domain representation (drawn to scale) of *C.elegans* (CeUNC-104). The different domains of *C. elegans* UNC-104 (as indicated from left to right in figure) are: Motor domain (aa 1-354), fork head-homology (FHA) domain (aa 463-592), homologous to liprin binding (LBD) region (aa 589-1267) and pleckstrin homology (PH) domain (aa 1460-1558). Details of mutations in the various alleles of *unc-104* are shown in the table below and their relative positions have been marked in the schematic representation. The intragenic suppressor that encodes UNC-104(D1497N M1540I) was isolated three independent times and named *sup1*, *tb101* and *tb120*. Of these the nucleotide change in *tb120* differs from those in *sup1* and *tb101* although the aa change is identical. (B) Primary sequence alignment of the PH domains of the following UNC-104 family members *C. elegans* (CeUNC-104), *Drosophila melanogaster* (DmUNC-104/*imac*), *H. sapiens* (HsATSV), *Mus musculus* (MmKIF1A) and *Dictostylium discoidum* (DdUNC-104). The D1497N residue mutated in *unc-104(e1265)* is highly conserved. The two intragenic suppressors *unc-104(e1265tb107)* and *unc-104(e1265tb120)* have two compensatory mutations M1540I and R1501Q respectively. The R1501 is well conserved while the M1540 varies but is still maintained as an acidic/neutral residue. Other residues demonstrated to be important for PI(4,5)P_2_ binding, KK1463/4, R1496 are also highlighted. In addition, another highly conserved residue W1549 that mediates the suppression of M1540I on N1497 has also been marked. (C) The RSCB protein data bank identifies DAPP1/PHISH (Dual adaptor of phosphotyrosine and 3-phosphoinositides, from *Homo sapiens*, PDB code 1FB8) as the closest homolog with an E-value of 3.4 [33]. The sequence identity and similarity between the query and templates were 22% and 38% respectively. * indicates identical amino acids, : indicates highly similar amino acids and . indicates similar amino acids between the DAPP1/PHISH and UNC-104 PH domains.
(4.41 MB TIF)

Figure S2 (A) Muscle arms are visualized using *trIs25*. Muscle arm number is altered in *unc-104(e1265)* as well as its suppressors. Muscle arm number is significantly decreased in *unc-104(e1265)* shown in (b) as compared to wild type (a) and partially restored in intragenic suppressors *unc-104(e1265tb107)* (c) and *unc-104(e1265tb120)* (d). The 9^th^ to 11^th^ muscles in the dorsal right quadrant are shown in all panels. Arrow points to muscles arms. Scale bar: 20 µm. (B) Quantitation of muscle arm numbers. Muscle arms are significantly reduced in *unc-104(e1265)*, but are partially restored in intragenic suppressors *unc-104(e1265tb107)* and *unc-104(e1265tb120)*. Data represented as mean ± SEM. *p<0.05 (C) Aldicarb paralysis assays of wild type, *unc-104(e1265), unc-104(e1265tb107)* and *unc-104(e1265tb120)* showing all time points assayed. (D) GFP::RAB-3 distribution in mechanosensory neurons using the transgenic line *jsIs821.* GFP::RAB-3 (pre-synaptic vesicle marker) distribution in NR and process of posterior lateral mechanosensory neuron (PLM process) shown respectively in wild type (a,b), *unc-104(e1265)* (c,d), *unc-104(e1265tb107*) (e,f), *unc-104(e1265tb120)* (g,h). When compared to *unc-104(e1265)* animals, increased signal resulting from greater transport was observed both in the NR and PLM processes of the suppressors. In (a, c, e, g) arrow points to the nerve ring and in PLM axon, the arrowhead and arrow mark the cell body and axon respectively. Scale bar: 10 μm. The alleles *unc-104(e1265)*, *unc-104(e1265tb107)* and *unc-104(e1265tb120)* are labeled in the figure by the respective protein changes they encode, namely D1497N, D1497N R1501Q and D1497N M1540I.
(3.08 MB TIF)

Figure S3 (A1,A2) Aldicarb paralysis/resistance assays in different mutant backgrounds that over-express *ppk-1* in neurons resulting in 40% increase in *in vivo* PI(4,5)P_2_ levels. (B1 and B2) Different transgenic variants of UNC-104::GFP (wild type, D/N, D/N R/Q, D/N M/I, D/N M/I W/A, M/I, R/Q, W/A) in an *unc-104(e1265)* background were tested for aldicarb analysis. We have shown data for two independently isolated transgenic lines for each UNC-104::GFP variant construct. (C1, C2) Different transgenic variants of UNC-104 lacking GFP (wt, D/N, D/N R/Q, D/N M/I) in an *unc-104(e1265)* background were tested for aldicarb analysis and locomotion . UNC-104 transgenes with and without GFP behave identically in these assays. (D) Quantitation of real time *unc-104* RNA levels in wild type and *unc-104(e1265)*. (n = 3 in duplicate). (E) Over expression of *ppk-1* using *gqIs125* also decreases the paralysis time in UNC-104(R1496A) and UNC-104(KK1463/4AA) transgenic lines. This demonstrates that the motors encoded by these transgenes are responsive to changes in PIP_2_ levels *in vivo* like *unc-104(e1265tb107)* and *unc-104(e1265tb120)*. Data represented as (mean ± SEM) time taken to paralyze the 50% of the worms. (n = 30). (F) Western blot analysis using anti-UNC-104 antibody of intragenic suppressors with and without *gqIs25* over expressing PI(4,5)P_2_ in neurons. Control for protein loading is done using an anti-tubulin antibody. The alleles *unc-104(e1265)*, *unc-104(e1265tb107)* and *unc-104(e1265tb120)* are labeled in the figure by the respective protein changes they encode, namely D1497N, D1497N R1501Q and D1497N M1540I.
(1.17 MB TIF)

Figure S4 Immunostaining of UNC-104 in wild type as well as different UNC-104 mutant alleles (A, B, D). (A) Immunostaining with anti-UNC-104 polyclonal antibody shows high immunoreactivity in (a) wild type as well as (b) *unc-104(rh43)* in nerve ring (shown by arrow) as compared to (c) *unc-104(e1265).* Arrow in B points to the cell body. Scale bar, 10µm. (B) Distribution of UNC-104 in wild type worms is pan-neurally expressed in synapse rich regions of the ventral cord and nerve ring (arrows), some commissural process and a few cell bodies near the nerve ring (arrowhead). Scale bar 10µm. (C) Expression of UNC-104::GFP with various PH domain mutations in the ventral cord, sub-lateral cords, commisures and dorsal cord. The UNC-104 motor with deletion of the PH domain sometimes lacks signal in the commisures (arrowhead). (D) In *unc-104(rh142)*; *jsIs682* (*unc-104* null mutant expressing GFP::RAB-3 pan-neurally) worms on which specificity of 25H11MAb against UNC-104 was tested. UNC-104 immunoreactivity was absent in (a) whereas immunoreactivity for GFP from GFP::RAB-3 was present (b) in worms of the same background. Scale bar 15µm. (E) Distribution of cargo (tagged with GFP::RAB-3) in the nerve ring and neuronal process of the PLM expressing UNC-104 PH domain variants lacking GFP in transgenic lines made in *unc-104(e1265); jsIs821* background. UNC-104 wild type protein (a, b). Intragenic mutant UNC-104 protein versions restore transport (e-h) while the UNC-104(D1497N) expressing transgene does not (c,d). Arrow indicates nerve ring (a,c,e,g) and PLM neuronal process (b,d,f,h). Arrowhead marks cell body of PLM neurons (b,d,f,h). Scale bar: 25µm. (F) Ratio of Mean fluorescence intensity (cell body/ synapse) of UNC-104::GFP with and without *uba-1(it129ts)* grown at 16 °C (permissive) and 22 °C (restrictive). (G, H) Ratio of fluorescent intensities in various parts of the PLM neuron in animals expressing UNC-104::GFP (*jsIs1111*), soluble GFP (*zdIs5*) in mechanosensory neurons. (I) anti-UNC-104 immunoreactivity in UNC-104 PH domain variant alleles over-expressing PI(4,5)P_2_ using the *gqIs25* transgene. Upon over-expressing PI(4,5)P_2_ UNC-104 levels consistently increase in the synapse-rich regions of the ventral cord of *unc-104(e1265tb107)* and *unc-104(e1265tb120)*. An occasional inconsistent increase was observed in *unc-104(e1265)* and no gross changes in a wild type UNC-104 background were observed. The alleles *unc-104(e1265)*, *unc-104(e1265tb107)* and *unc-104(e1265tb120)* are labeled in the figure by the respective protein changes they encode, namely D1497N, D1497N R1501Q and D1497N M1540I.
(1.99 MB TIF)

Video S1 Transport of GFP::RAB-3 in the anterior mechanosensory neurons of different genotypes.
Movement of GFP::RAB-3 in wild type neurons.
(3.09 MB MOV)

Video S2 Movement of GFP::RAB-3 marked vesicles in *unc-104(e1265).*
(1.58 MB MOV)

Video S3 Movement of GFP::RAB-3 in *unc-104(e1265tb120).*
(1.08 MB MOV)
