# Supplementary figures and images for: The Caenorhabditis elegans Kinesin-3 Motor UNC-104/KIF1A Is Degraded upon Loss of Specific Binding to Cargo
Source: PLoS Genet. 2010 Nov 4;6(11):e1001200. doi: 10.1371/journal.pgen.1001200 (PMC2973836; doi:10.1371/journal.pgen.1001200)

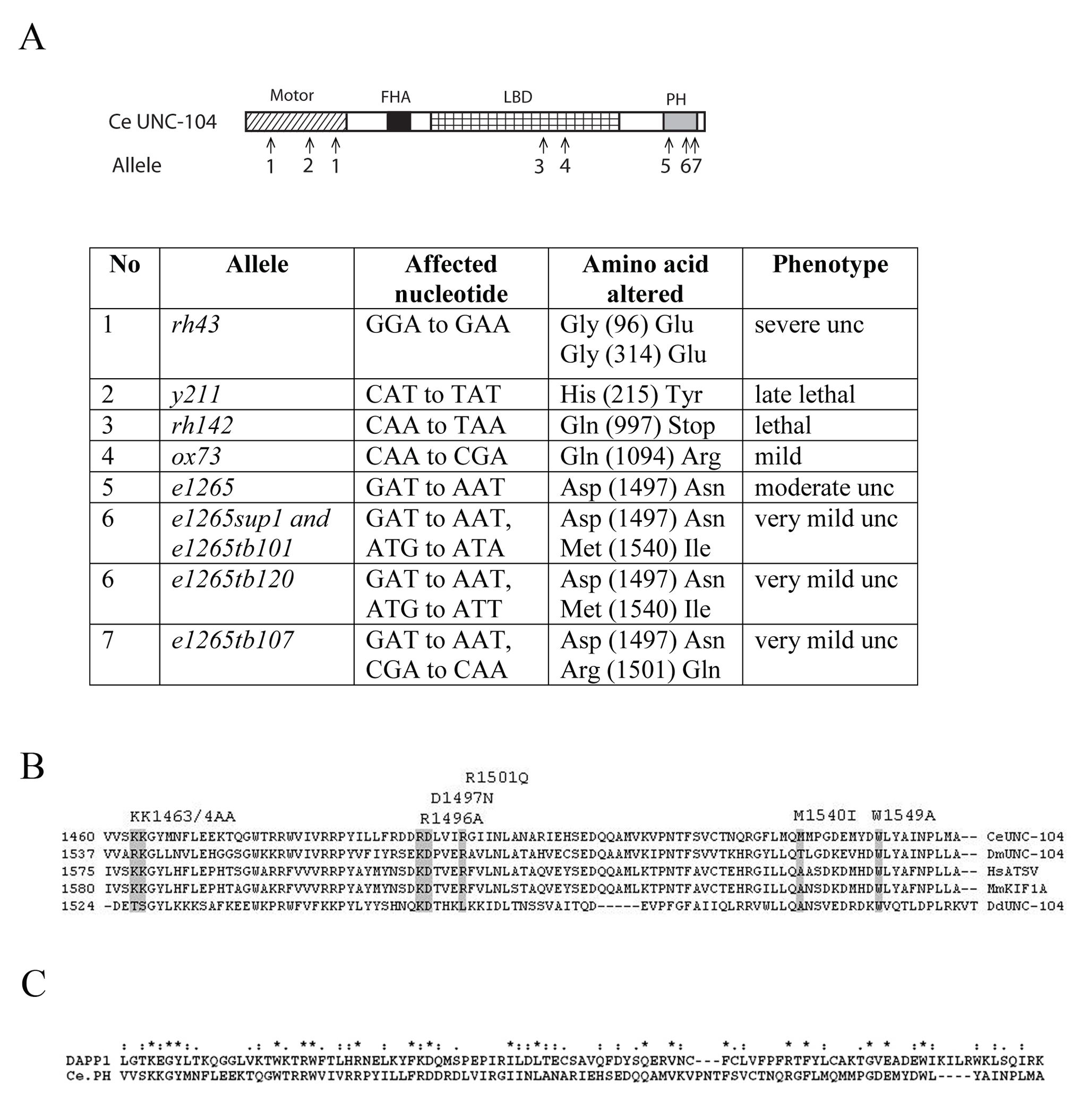

Supplement: Figure S1 — (A) A schematic domain representation (drawn to scale) of C.elegans (CeUNC-104). The different domains of C. elegans UNC-104 (as indicated from left to right in figure) are: Motor domain (aa 1-354), fork head-homology (FHA) domain (aa 463-592), homologous to liprin binding (LBD) region (aa 589-1267) and pleckstrin homology (PH) domain (aa 1460-1558). Details of mutations in the various alleles of unc-104 are shown in the table below and their relative positions have been marked in the schematic representation. The intragenic suppressor that encodes UNC-104(D1497N M1540I) was isolated three independent times and named sup1, tb101 and tb120. Of these the nucleotide change in tb120 differs from those in sup1 and tb101 although the aa change is identical. (B) Primary sequence alignment of the PH domains of the following UNC-104 family members C. elegans (CeUNC-104), Drosophila melanogaster (DmUNC-104/imac), H. sapiens (HsATSV), Mus musculus (MmKIF1A) and Dictostylium discoidum (DdUNC-104). The D1497N residue mutated in unc-104(e1265) is highly conserved. The two intragenic suppressors unc-104(e1265tb107) and unc-104(e1265tb120) have two compensatory mutations M1540I and R1501Q respectively. The R1501 is well conserved while the M1540 varies but is still maintained as an acidic/neutral residue. Other residues demonstrated to be important for PI(4,5)P2 binding, KK1463/4, R1496 are also highlighted. In addition, another highly conserved residue W1549 that mediates the suppression of M1540I on N1497 has also been marked. (C) The RSCB protein data bank identifies DAPP1/PHISH (Dual adaptor of phosphotyrosine and 3-phosphoinositides, from Homo sapiens, PDB code 1FB8) as the closest homolog with an E-value of 3.4 [33]. The sequence identity and similarity between the query and templates were 22% and 38% respectively. * indicates identical amino acids, : indicates highly similar amino acids and . indicates similar amino acids between the DAPP1/PHISH and UNC-104 PH domains. (4.41 [file pgen.1001200.s001.tif]

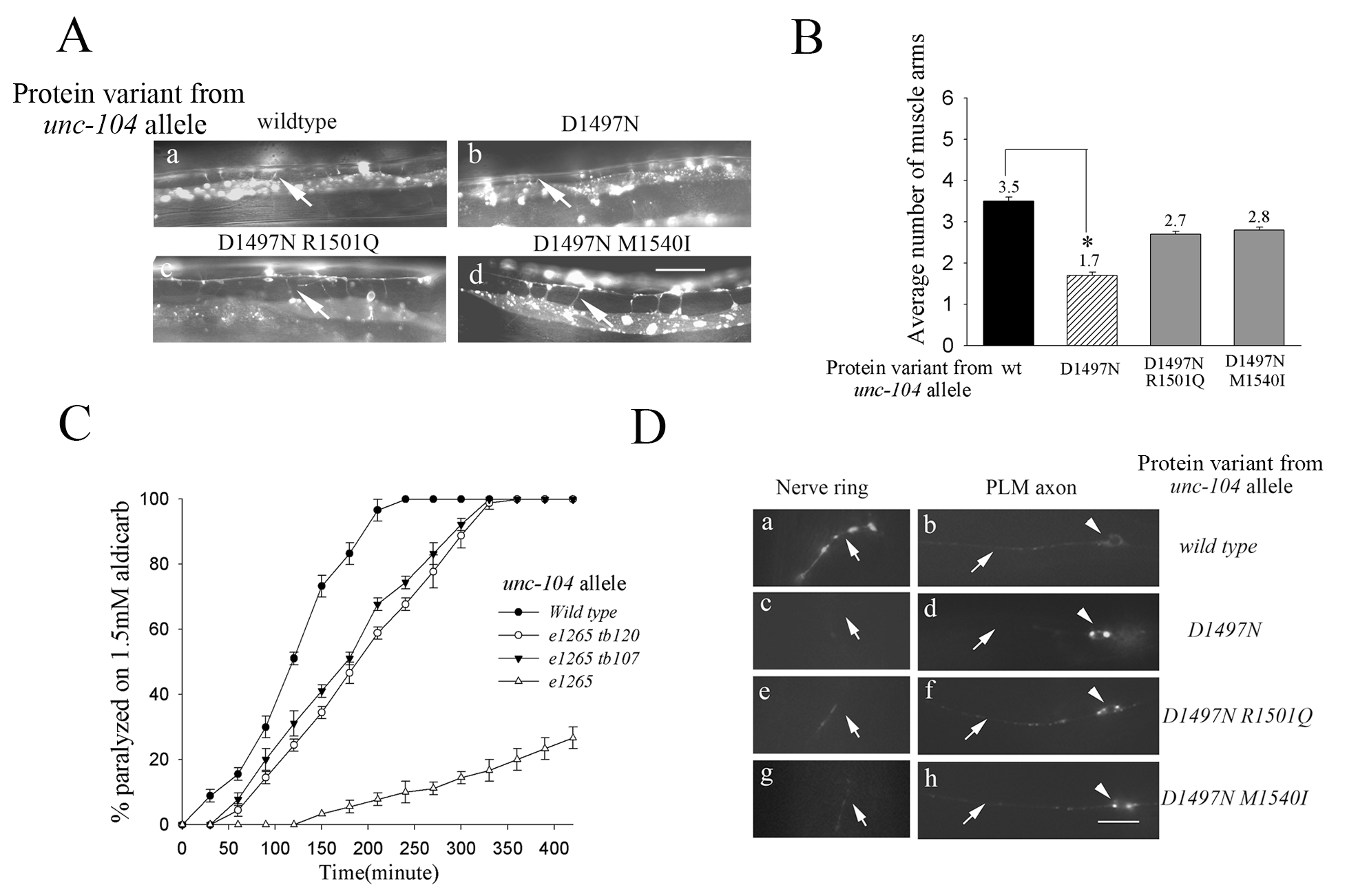

Supplement: Figure S2 — (A) Muscle arms are visualized using trIs25. Muscle arm number is altered in unc-104(e1265) as well as its suppressors. Muscle arm number is significantly decreased in unc-104(e1265) shown in (b) as compared to wild type (a) and partially restored in intragenic suppressors unc-104(e1265tb107) (c) and unc-104(e1265tb120) (d). The 9th to 11th muscles in the dorsal right quadrant are shown in all panels. Arrow points to muscles arms. Scale bar: 20 µm. (B) Quantitation of muscle arm numbers. Muscle arms are significantly reduced in unc-104(e1265), but are partially restored in intragenic suppressors unc-104(e1265tb107) and unc-104(e1265tb120). Data represented as mean ± SEM. *p<0.05 (C) Aldicarb paralysis assays of wild type, unc-104(e1265), unc-104(e1265tb107) and unc-104(e1265tb120) showing all time points assayed. (D) GFP::RAB-3 distribution in mechanosensory neurons using the transgenic line jsIs821. GFP::RAB-3 (pre-synaptic vesicle marker) distribution in NR and process of posterior lateral mechanosensory neuron (PLM process) shown respectively in wild type (a,b), unc-104(e1265) (c,d), unc-104(e1265tb107) (e,f), unc-104(e1265tb120) (g,h). When compared to unc-104(e1265) animals, increased signal resulting from greater transport was observed both in the NR and PLM processes of the suppressors. In (a, c, e, g) arrow points to the nerve ring and in PLM axon, the arrowhead and arrow mark the cell body and axon respectively. Scale bar: 10 μm. The alleles unc-104(e1265), unc-104(e1265tb107) and unc-104(e1265tb120) are labeled in the figure by the respective protein changes they encode, namely D1497N, D1497N R1501Q and D1497N M1540I. (3.08 MB TIF) [file pgen.1001200.s002.tif]

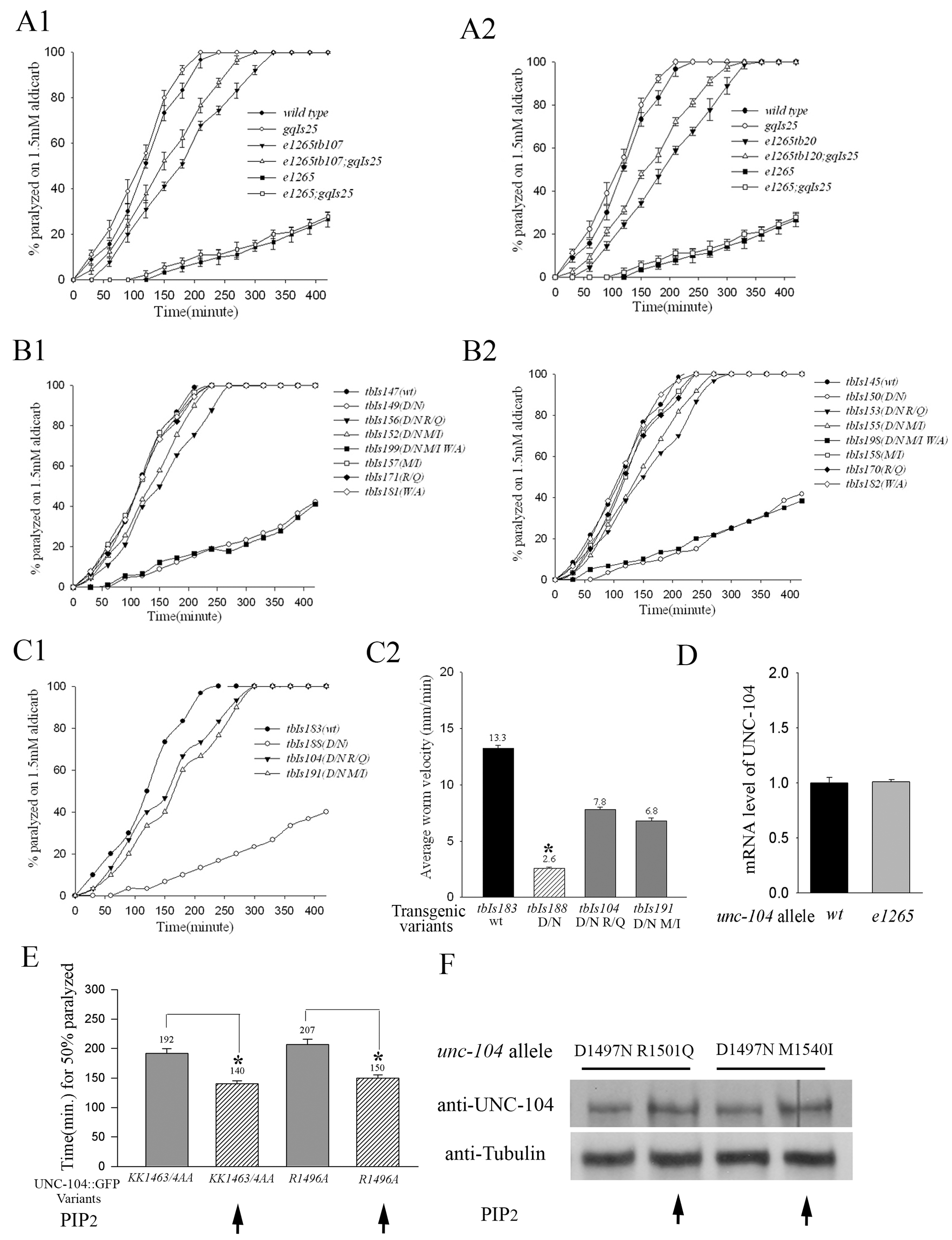

Supplement: Figure S3 — (A1,A2) Aldicarb paralysis/resistance assays in different mutant backgrounds that over-express ppk-1 in neurons resulting in 40% increase in in vivo PI(4,5)P2 levels. (B1 and B2) Different transgenic variants of UNC-104::GFP (wild type, D/N, D/N R/Q, D/N M/I, D/N M/I W/A, M/I, R/Q, W/A) in an unc-104(e1265) background were tested for aldicarb analysis. We have shown data for two independently isolated transgenic lines for each UNC-104::GFP variant construct. (C1, C2) Different transgenic variants of UNC-104 lacking GFP (wt, D/N, D/N R/Q, D/N M/I) in an unc-104(e1265) background were tested for aldicarb analysis and locomotion . UNC-104 transgenes with and without GFP behave identically in these assays. (D) Quantitation of real time unc-104 RNA levels in wild type and unc-104(e1265). (n = 3 in duplicate). (E) Over expression of ppk-1 using gqIs125 also decreases the paralysis time in UNC-104(R1496A) and UNC-104(KK1463/4AA) transgenic lines. This demonstrates that the motors encoded by these transgenes are responsive to changes in PIP2 levels in vivo like unc-104(e1265tb107) and unc-104(e1265tb120). Data represented as (mean ± SEM) time taken to paralyze the 50% of the worms. (n = 30). (F) Western blot analysis using anti-UNC-104 antibody of intragenic suppressors with and without gqIs25 over expressing PI(4,5)P2 in neurons. Control for protein loading is done using an anti-tubulin antibody. The alleles unc-104(e1265), unc-104(e1265tb107) and unc-104(e1265tb120) are labeled in the figure by the respective protein changes they encode, namely D1497N, D1497N R1501Q and D1497N M1540I. (1.17 MB TIF) [file pgen.1001200.s003.tif]

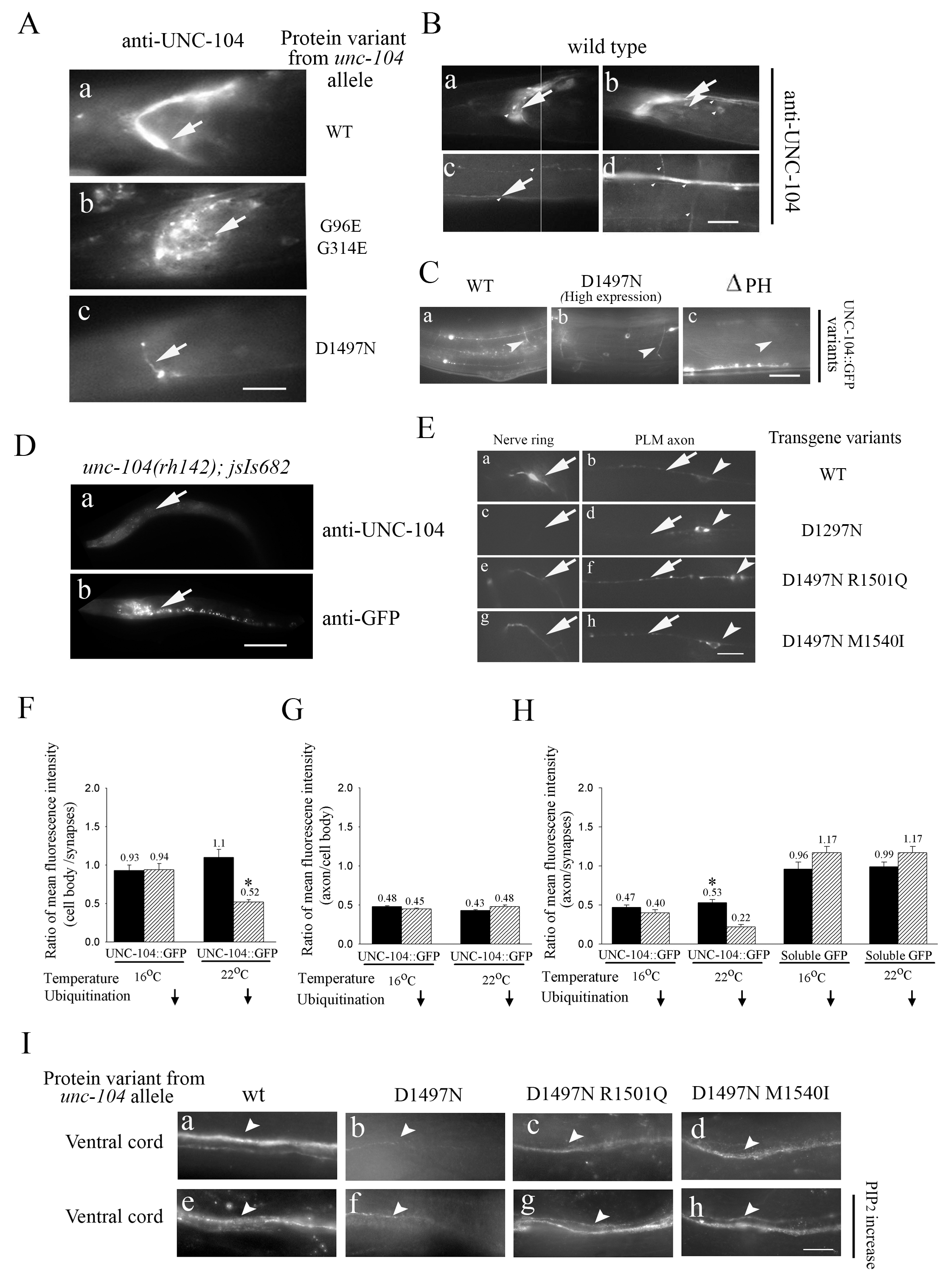

Supplement: Figure S4 — Immunostaining of UNC-104 in wild type as well as different UNC-104 mutant alleles (A, B, D). (A) Immunostaining with anti-UNC-104 polyclonal antibody shows high immunoreactivity in (a) wild type as well as (b) unc-104(rh43) in nerve ring (shown by arrow) as compared to (c) unc-104(e1265). Arrow in B points to the cell body. Scale bar, 10µm. (B) Distribution of UNC-104 in wild type worms is pan-neurally expressed in synapse rich regions of the ventral cord and nerve ring (arrows), some commissural process and a few cell bodies near the nerve ring (arrowhead). Scale bar 10µm. (C) Expression of UNC-104::GFP with various PH domain mutations in the ventral cord, sub-lateral cords, commisures and dorsal cord. The UNC-104 motor with deletion of the PH domain sometimes lacks signal in the commisures (arrowhead). (D) In unc-104(rh142); jsIs682 (unc-104 null mutant expressing GFP::RAB-3 pan-neurally) worms on which specificity of 25H11MAb against UNC-104 was tested. UNC-104 immunoreactivity was absent in (a) whereas immunoreactivity for GFP from GFP::RAB-3 was present (b) in worms of the same background. Scale bar 15µm. (E) Distribution of cargo (tagged with GFP::RAB-3) in the nerve ring and neuronal process of the PLM expressing UNC-104 PH domain variants lacking GFP in transgenic lines made in unc-104(e1265); jsIs821 background. UNC-104 wild type protein (a, b). Intragenic mutant UNC-104 protein versions restore transport (e-h) while the UNC-104(D1497N) expressing transgene does not (c,d). Arrow indicates nerve ring (a,c,e,g) and PLM neuronal process (b,d,f,h). Arrowhead marks cell body of PLM neurons (b,d,f,h). Scale bar: 25µm. (F) Ratio of Mean fluorescence intensity (cell body/ synapse) of UNC-104::GFP with and without uba-1(it129ts) grown at 16 °C (permissive) and 22 °C (restrictive). (G, H) Ratio of fluorescent intensities in various parts of the PLM neuron in animals expressing UNC-104::GFP (jsIs1111), soluble GFP (zdIs5) in mechanosensory neurons. (I) anti-UNC-104 i [file pgen.1001200.s004.tif]
